# Supplementary material for: Local translatome sustains synaptic function in impaired Wallerian degeneration
Source: EMBO Rep. 2024 Oct 31;26(1):61–83. doi: 10.1038/s44319-024-00301-8 (PMC11724096; doi:10.1038/s44319-024-00301-8)
Supplement: Supplementary file 3 — Appendix [file 44319_2024_301_MOESM3_ESM.pdf]

Appendix

Local translatome sustains synaptic function in impaired Wallerian degeneration

Maria Paglione[1,+], Leonardo Restivo[1], Sarah Zakhia[2], Arnau Llobet Rosell[1], Marco Terenzio[2] and Lukas J. Neukomm[1,*]

[1] Department of Fundamental Neurosciences, University of Lausanne, 1005 Lausanne, Switzerland.

[2] Molecular Neuroscience Unit, Okinawa Institute of Science and Technology Graduate University, Kunigami-gun, Okinawa 904-0412, Japan.

[+] Lemanic Neuroscience Doctoral School (LNDS).

* Correspondence:
lukas.neukomm@unil.ch
+41 21 692 52 88

Table of content

Appendix Figure S1.	Page 3
Appendix Figure S2.	Page 4
Appendix Figure S3.	Page 5
Appendix Figure S4.	Page 6

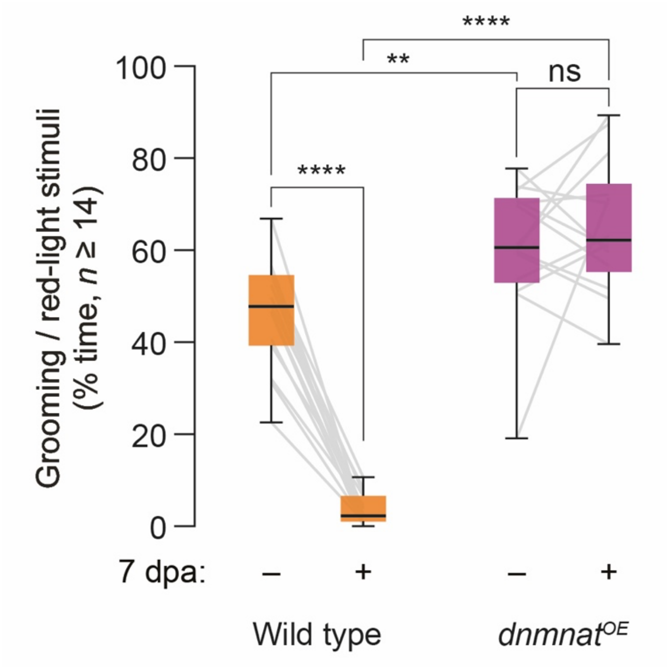

Appendix Figure S1. Evoked antennal grooming mediated by CsChrimson expressed from the X chromosome. Preservation of synaptic function in severed *dnmnat*^{OE} projections at 7 dpa. Grooming in uninjured and injured animals (% time of red-light stimuli; $n \geq 14$ animals). Two-way ANOVA with Sidak's multiple comparisons test; ****, $p < 0.0001$; **, $p < 0.01$.

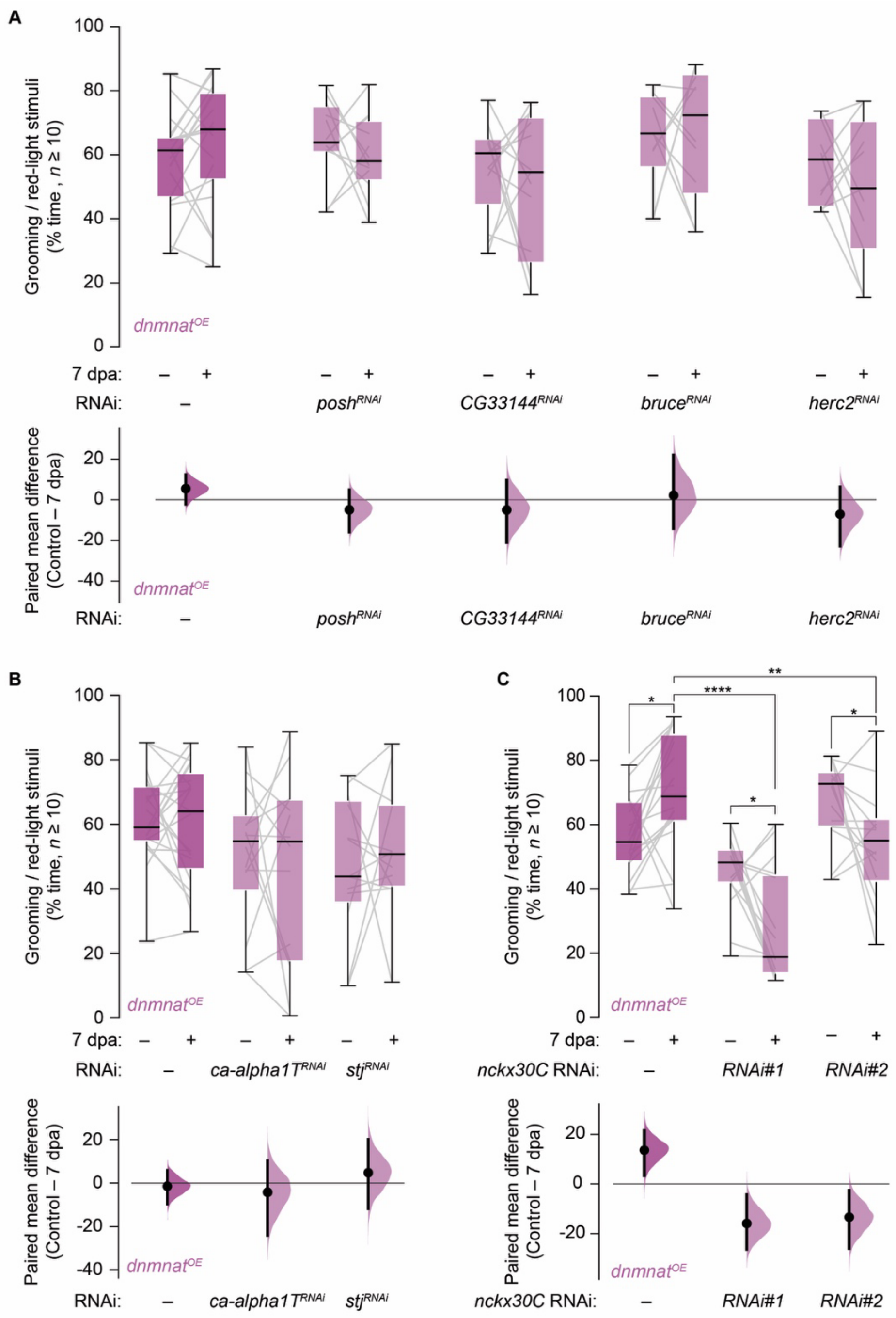

Appendix Figure S2. Protein ubiquitination and calcium transport candidate screen. A Top, protein ubiquitination candidate genes (Grooming as % time of red-light stimuli). Bottom, paired mean difference as bootstrap sampling distribution (dot, mean difference; vertical error bar, 95 % confidence interval, respectively; $n \geq 10$ animals). **B** Top, Ca^{2+} transport candidate genes; bottom, paired mean difference ($n \geq 10$ animals). **C** Top, validation of *nckx30C* by two different RNAi lines (#1 and #2, respectively); bottom paired mean difference ($n \geq 10$ animals). Paired two-tailed t-student and Two-Way ANOVA with Dunnett's multiple comparisons test; ****, $p < 0.0001$; **, $p < 0.01$; *, $p < 0.05$.

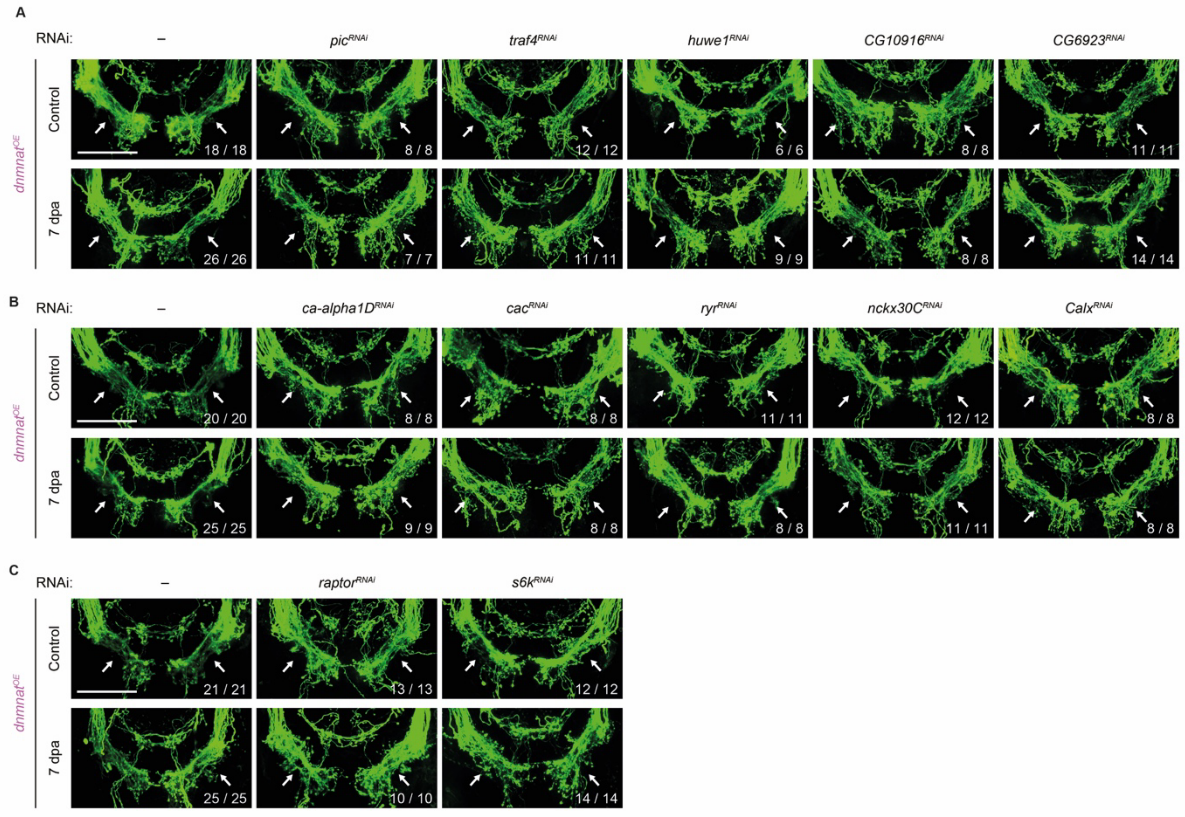

Appendix Figure S3. Preserved axonal and synaptic morphology in RNAi-mediated candidate knockdown one week after axotomy. A Examples of GFP-labeled *dnmnat*^{OE} *JO*$^{+}$ mechanosensory axonal projections with protein ubiquitination *candidate*^{RNAi}. **B** Examples of GFP-labeled *dnmnat*^{OE} *JO*$^{+}$ mechanosensory axonal projections with Ca^{2+} transport *candidate*^{RNAi}. **C** Examples of GFP-labeled *dnmnat*^{OE} *JO*$^{+}$ mechanosensory axonal projections with *raptor*^{RNAi} and *s6k*^{RNAi}*.* Top and bottom, control and 7 dpa, respectively. Arrows, projections; quantification, brains with preserved *JO*$^{+}$ projections / total brains analyzed (*n,* bottom right corner). Scale bar, 50 µm.

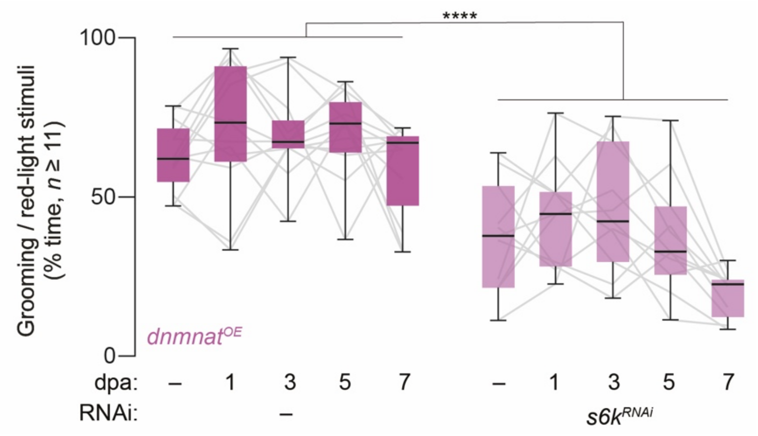

Appendix Figure S4. RNAi-mediated knockdown of *s6k* progressively reduces preserved synaptic function after axotomy. Grooming as % time of red-light stimuli ($n \geq 11$ animals). Two-way ANOVA; ****, $p < 0.0001$.
