# Supplementary figures and images for: Local translatome sustains synaptic function in impaired Wallerian degeneration
Source: EMBO Rep. 2024 Oct 31;26(1):61–83. doi: 10.1038/s44319-024-00301-8 (PMC11724096; doi:10.1038/s44319-024-00301-8)

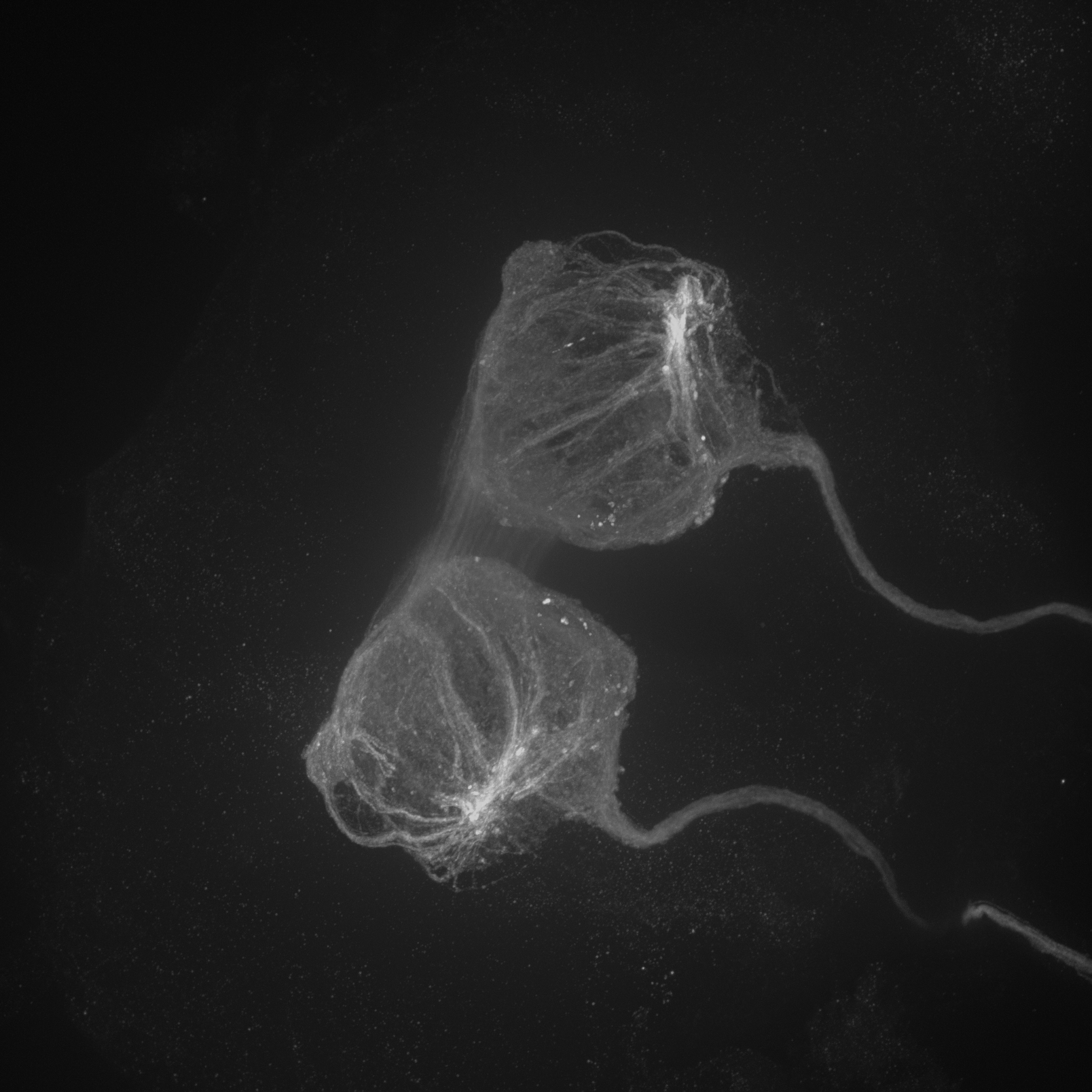

Supplement: Supplementary file 15 — Source data Fig. 1 [file 44319_2024_301_MOESM15_ESM.zip › Figure 1/1H/GFP-MAX_orco_nmnat_14dpi_3.tif]

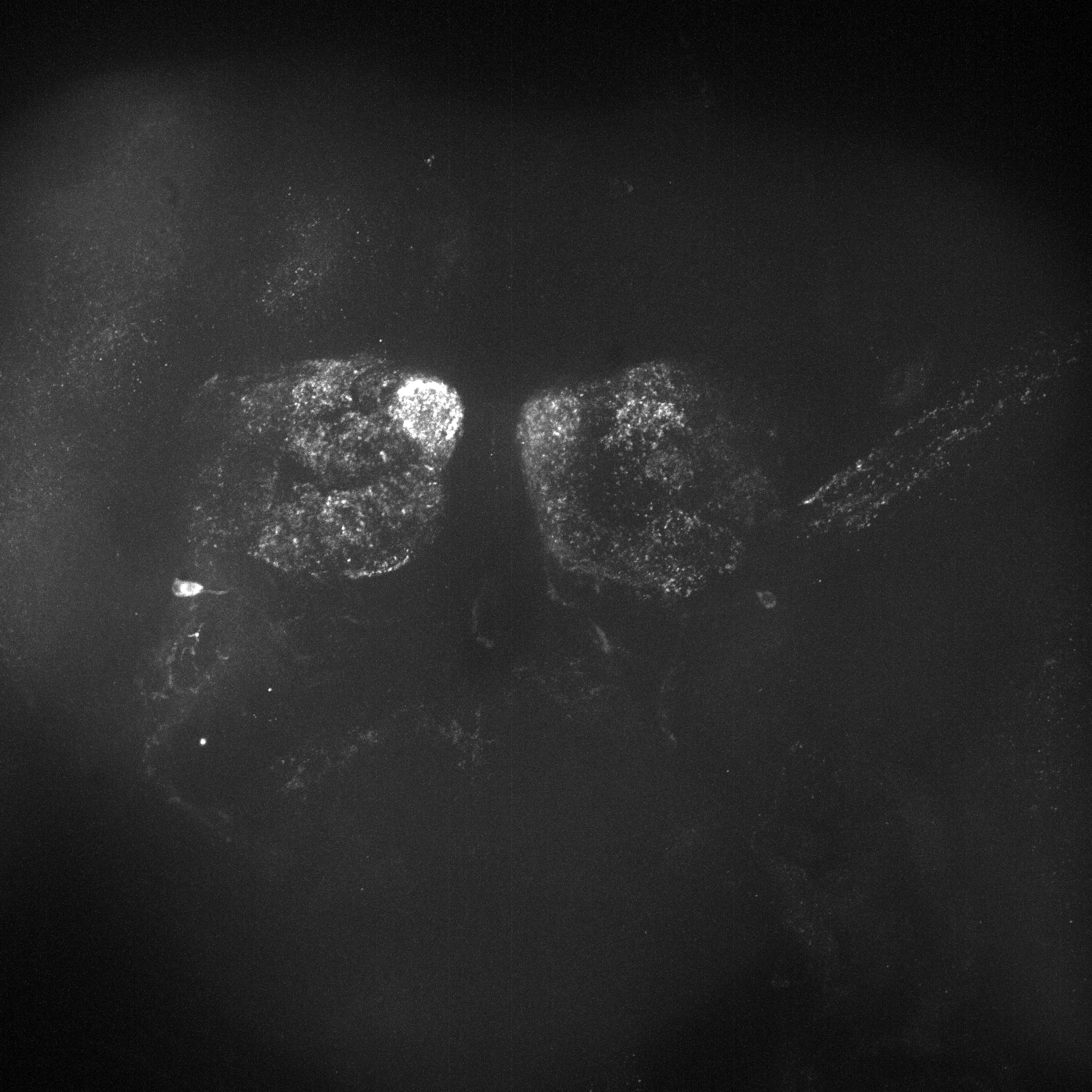

Supplement: Supplementary file 15 — Source data Fig. 1 [file 44319_2024_301_MOESM15_ESM.zip › Figure 1/1H/GFP-orco_wt_14_dpi_2_043-MaxIP.tif]

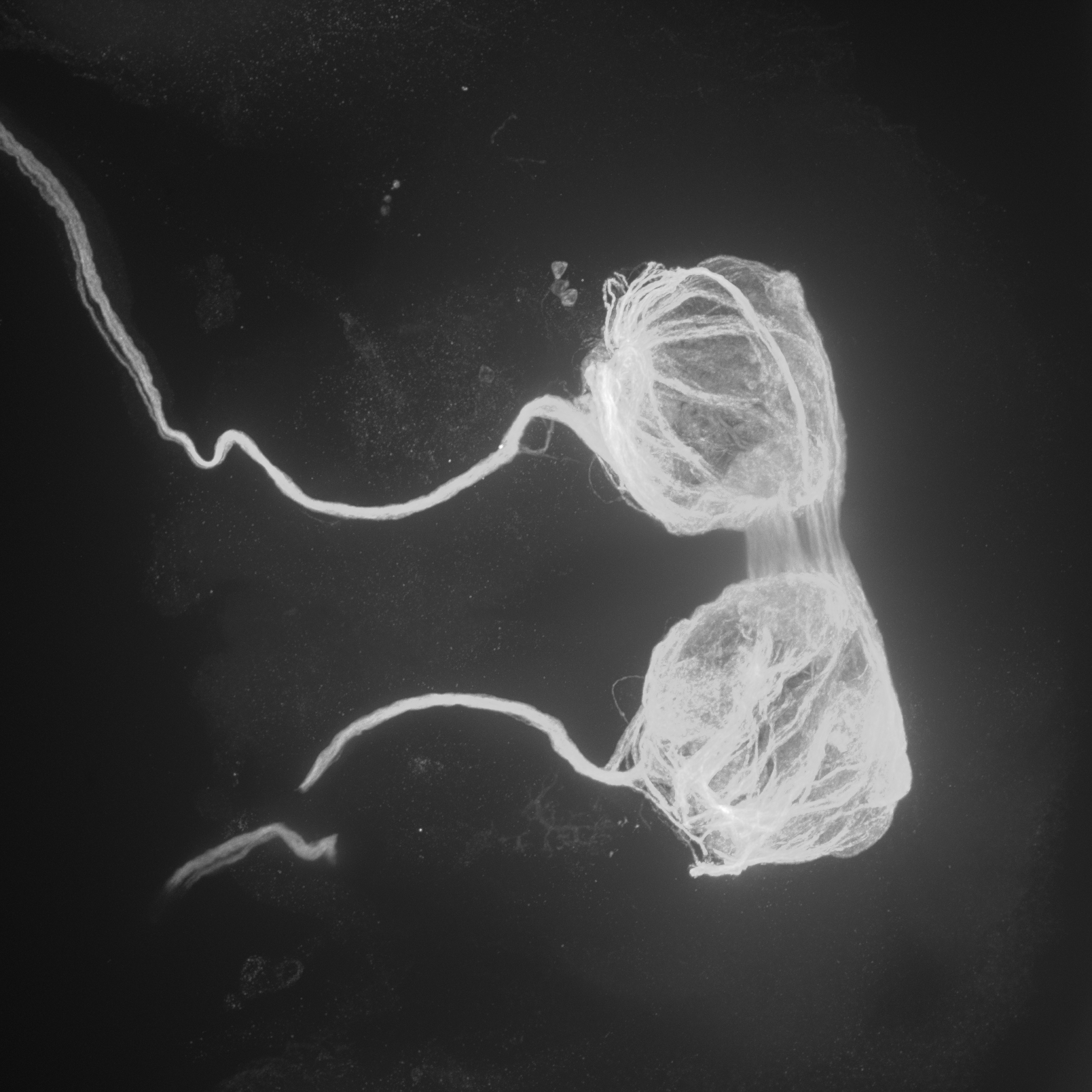

Supplement: Supplementary file 15 — Source data Fig. 1 [file 44319_2024_301_MOESM15_ESM.zip › Figure 1/1H/GFP-MAX_orco_wt_bef_7_#5.tif]

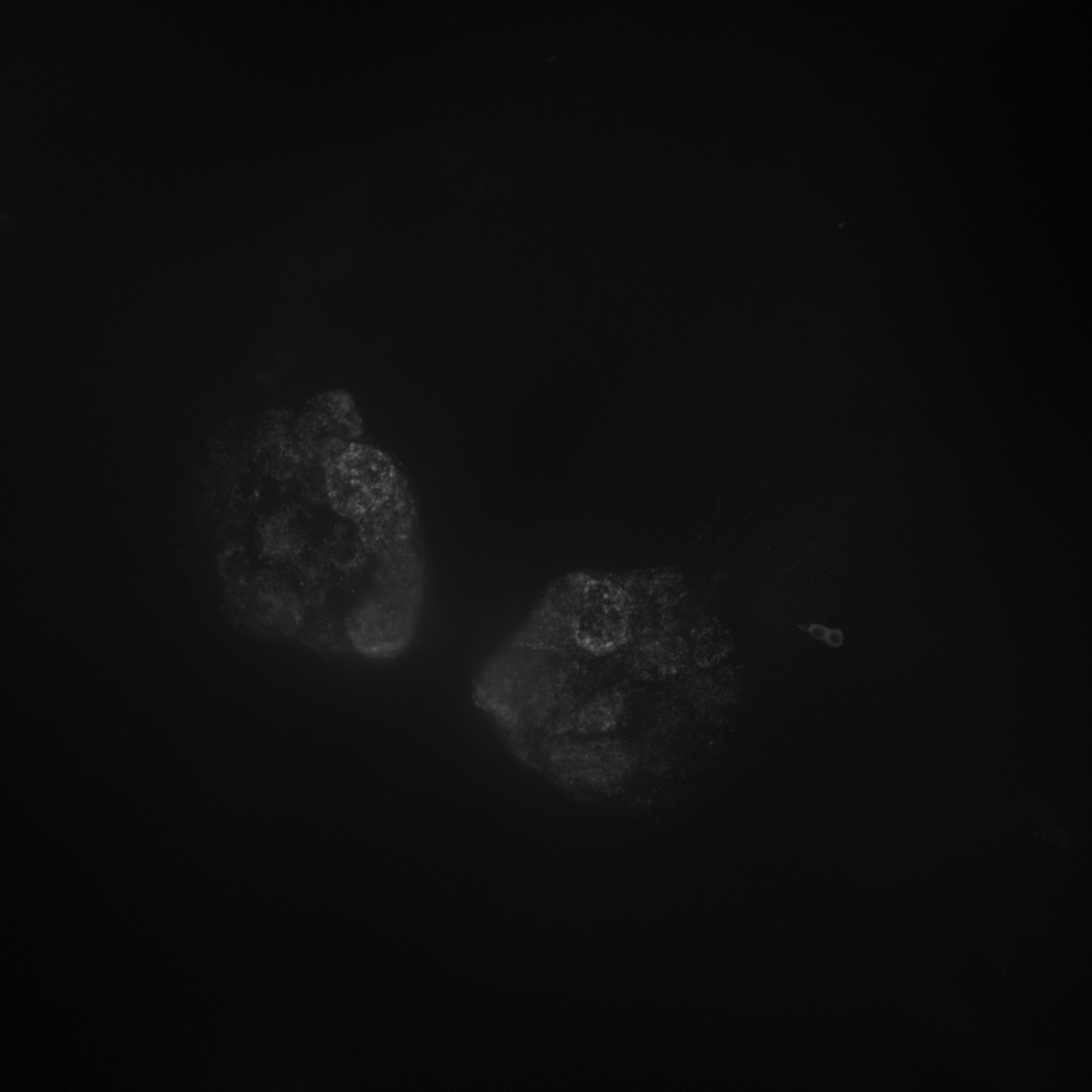

Supplement: Supplementary file 15 — Source data Fig. 1 [file 44319_2024_301_MOESM15_ESM.zip › Figure 1/1H/GFP-MAX_orco_wt_7dpi_1_062.tif]

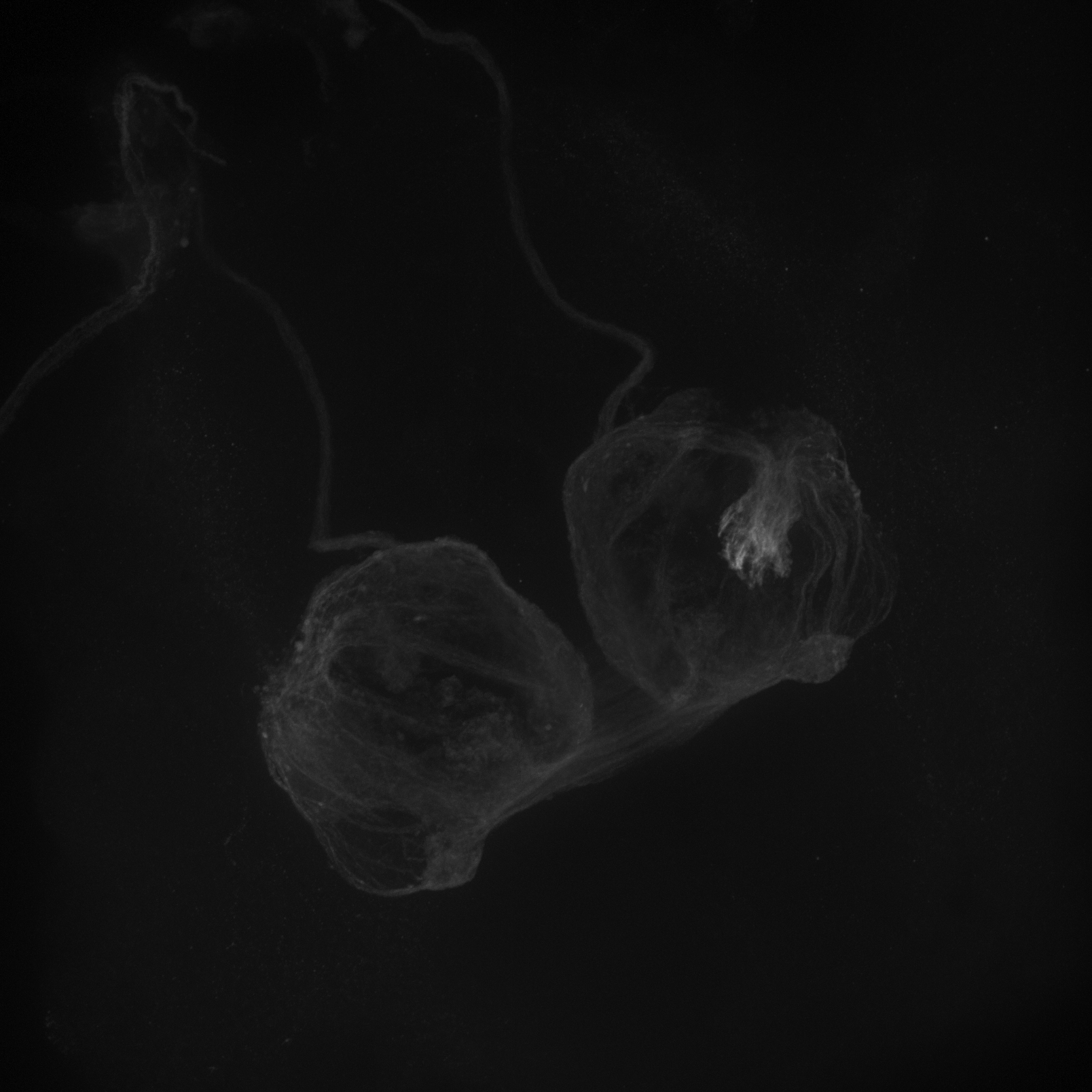

Supplement: Supplementary file 15 — Source data Fig. 1 [file 44319_2024_301_MOESM15_ESM.zip › Figure 1/1H/GFP-MAX_orco_nmnat_7_dpi_6_107.tif]

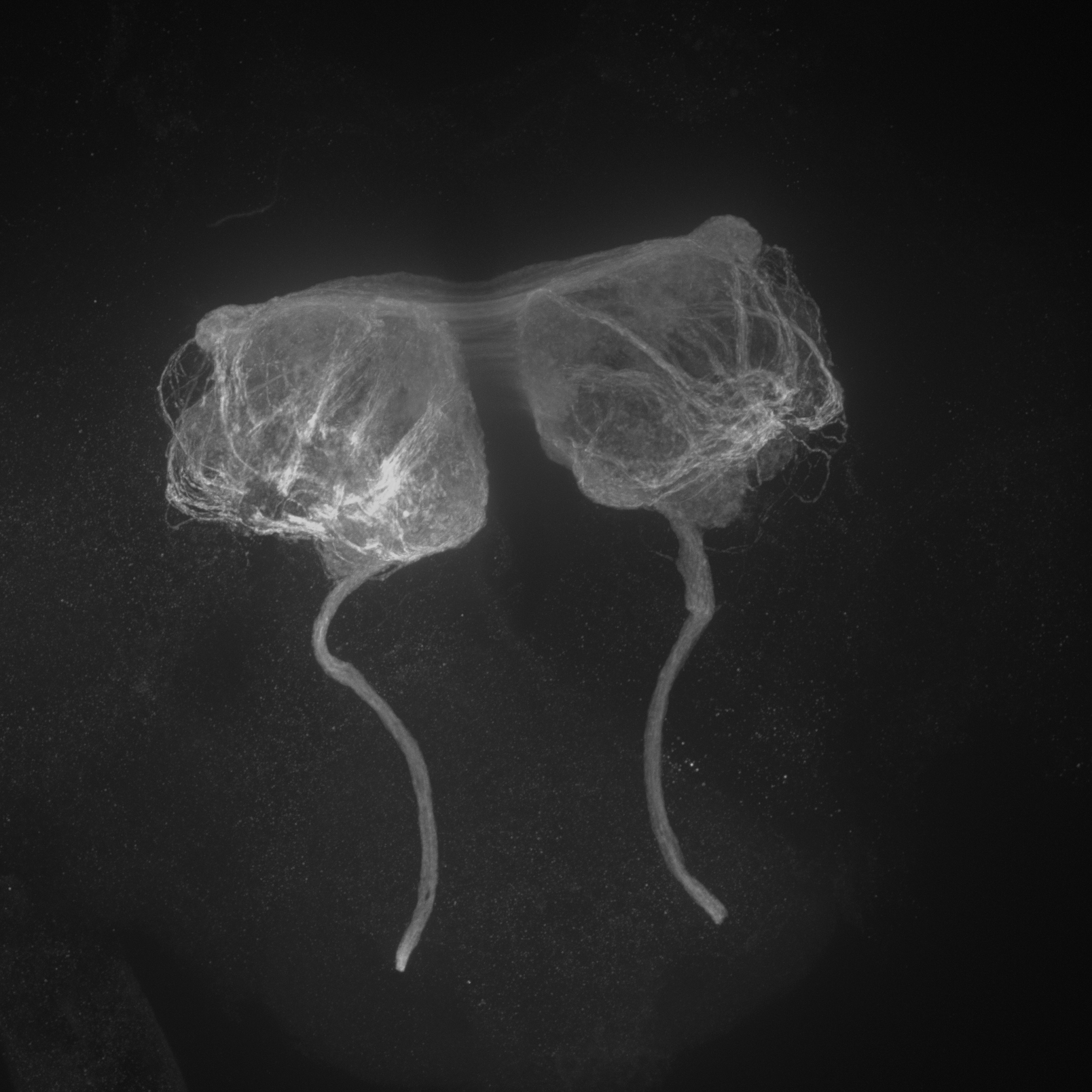

Supplement: Supplementary file 15 — Source data Fig. 1 [file 44319_2024_301_MOESM15_ESM.zip › Figure 1/1H/GFP-MAX_nmnat_orco_before5.001_IIexp.tif]

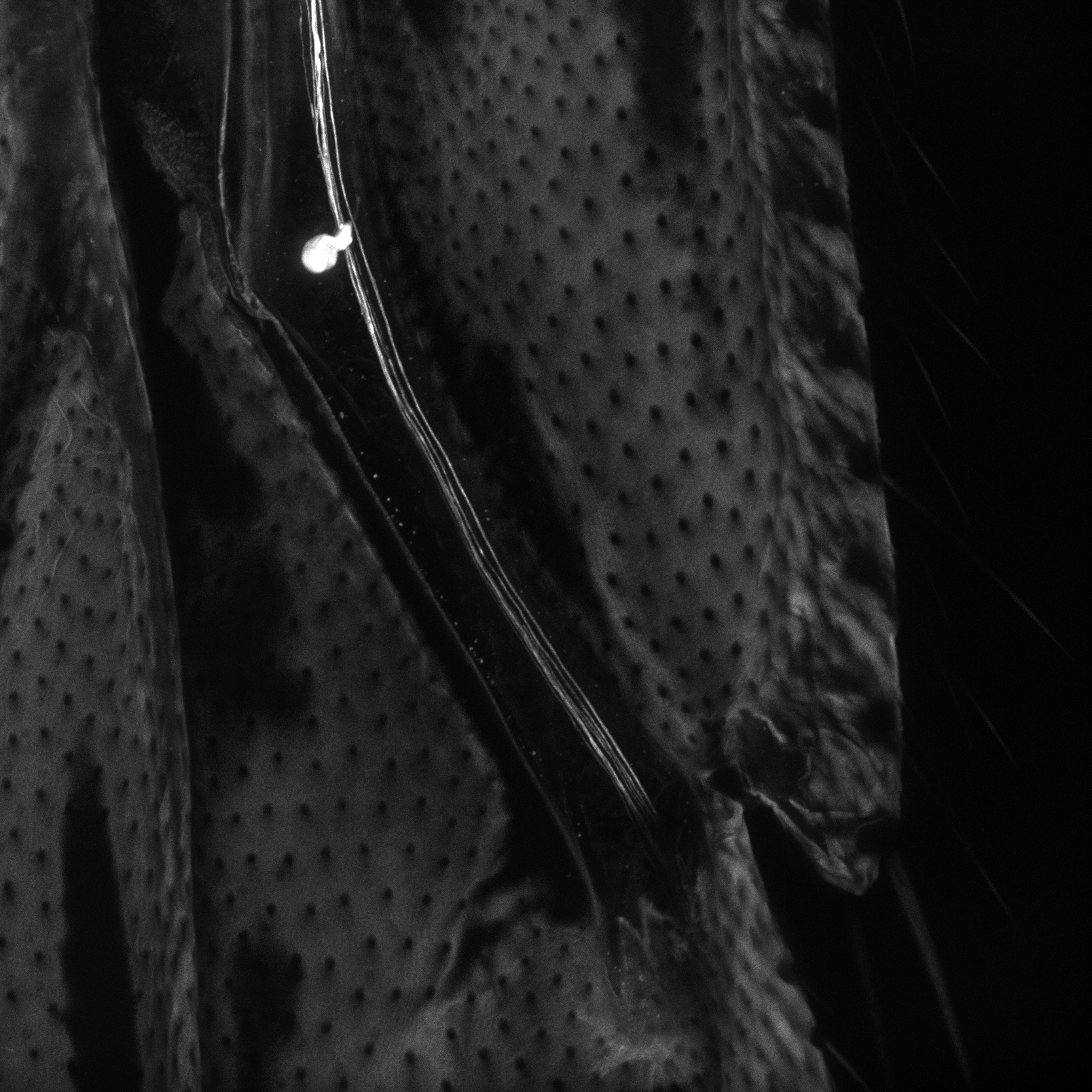

Supplement: Supplementary file 15 — Source data Fig. 1 [file 44319_2024_301_MOESM15_ESM.zip › Figure 1/1A/control nmnat before injury.tif]

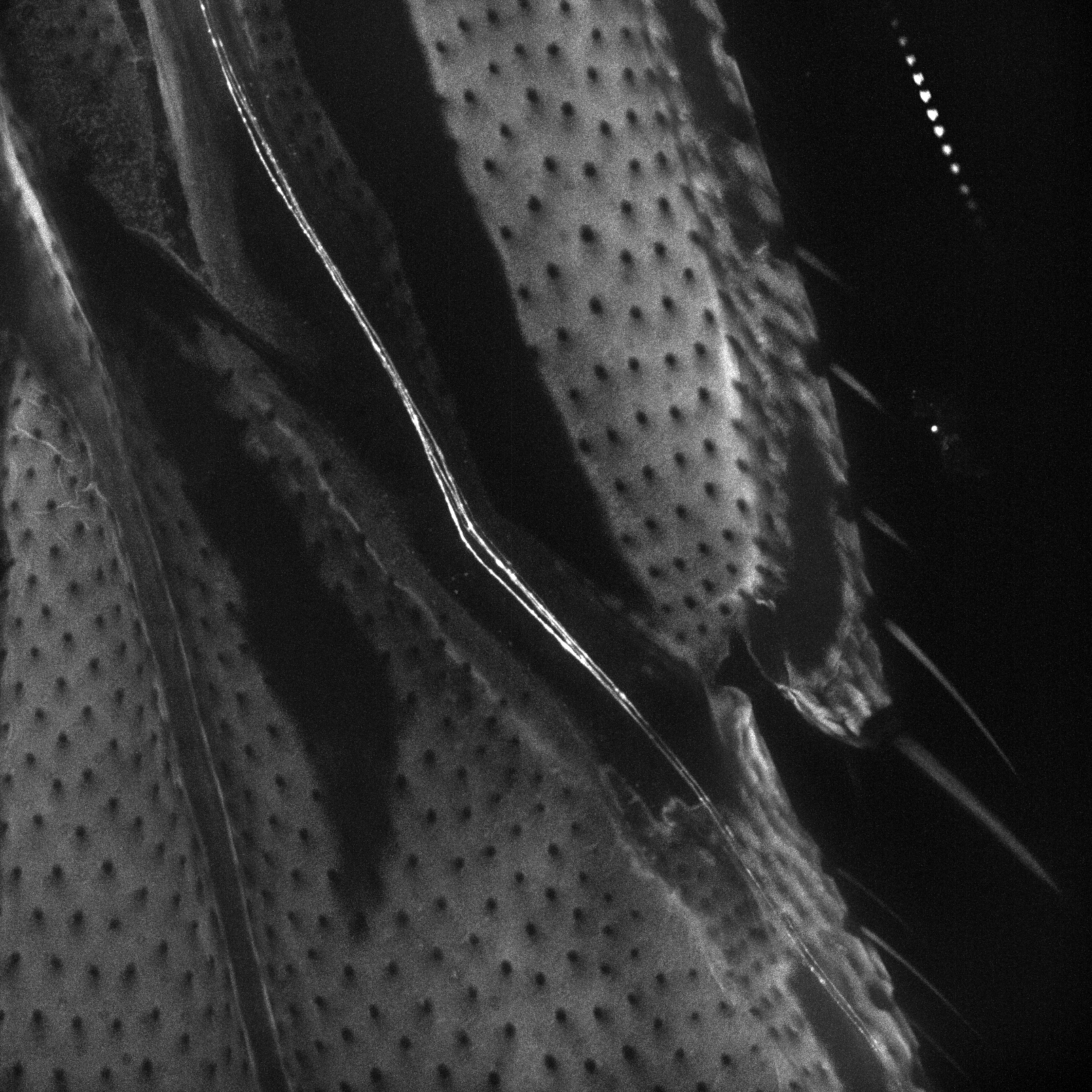

Supplement: Supplementary file 15 — Source data Fig. 1 [file 44319_2024_301_MOESM15_ESM.zip › Figure 1/1A/nmnatoe 2 week after injury.tif]

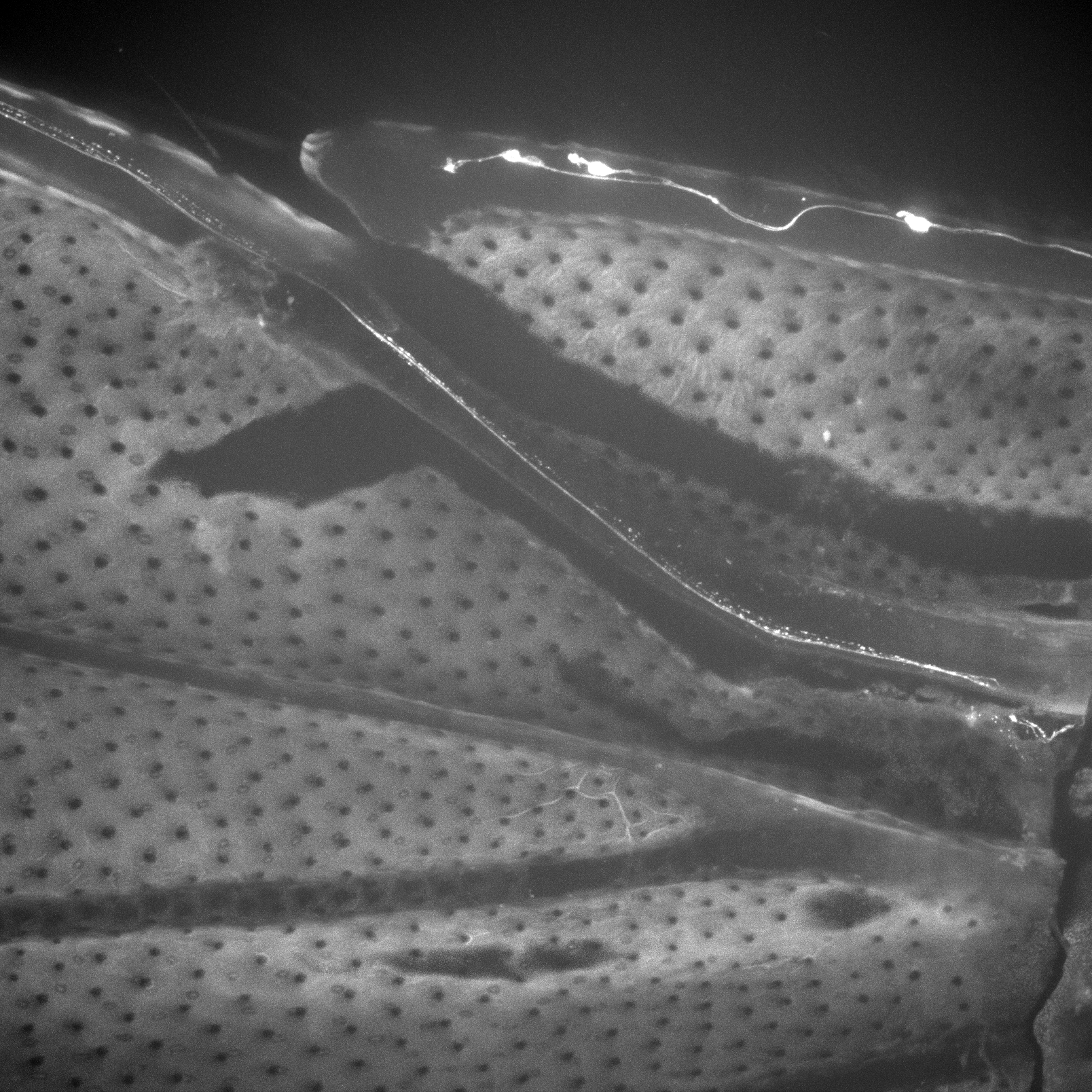

Supplement: Supplementary file 15 — Source data Fig. 1 [file 44319_2024_301_MOESM15_ESM.zip › Figure 1/1A/injured wt 1 week after injury.tif]

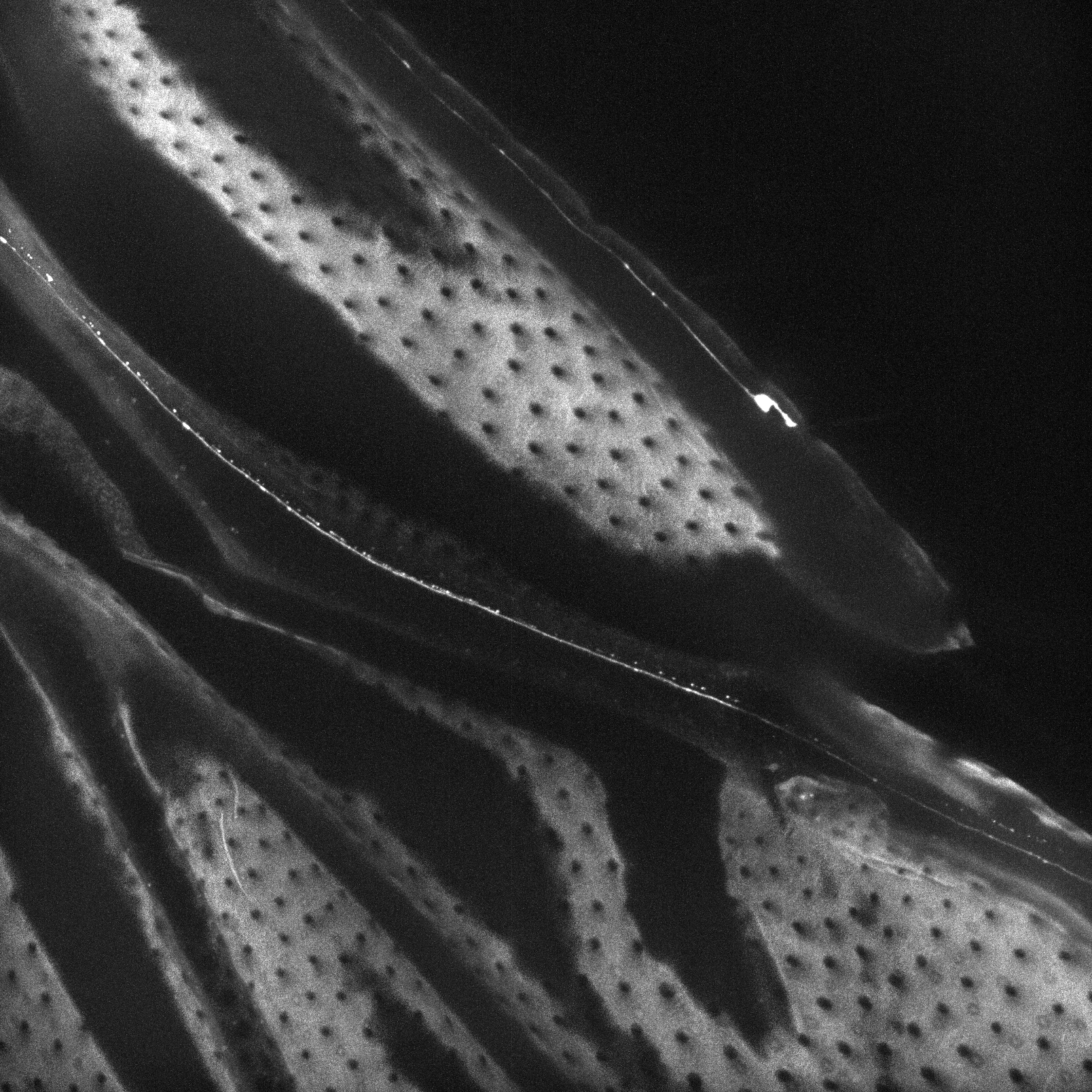

Supplement: Supplementary file 15 — Source data Fig. 1 [file 44319_2024_301_MOESM15_ESM.zip › Figure 1/1A/injured wt 2 weeks after injury.tif]

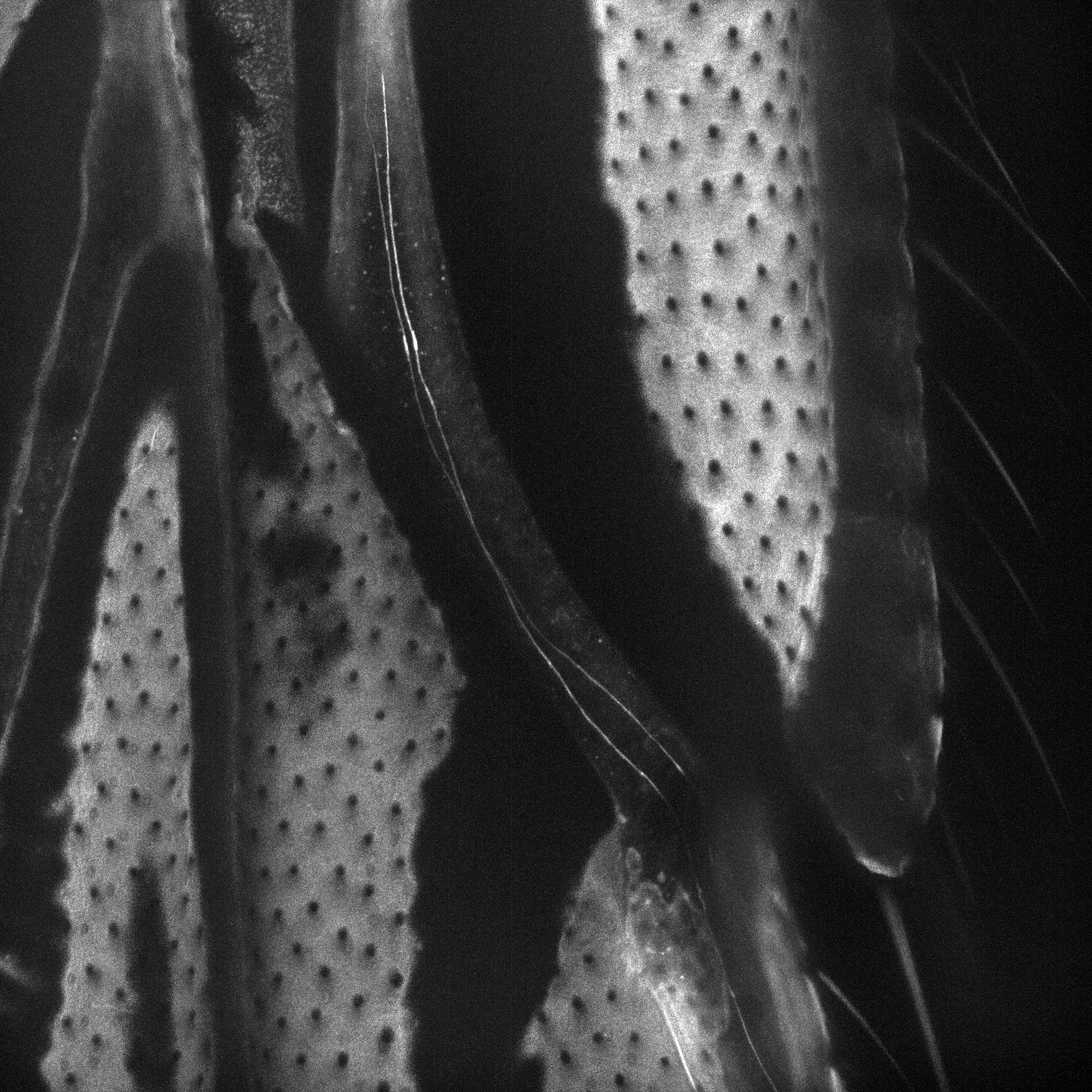

Supplement: Supplementary file 15 — Source data Fig. 1 [file 44319_2024_301_MOESM15_ESM.zip › Figure 1/1A/control wt before injury.tif]

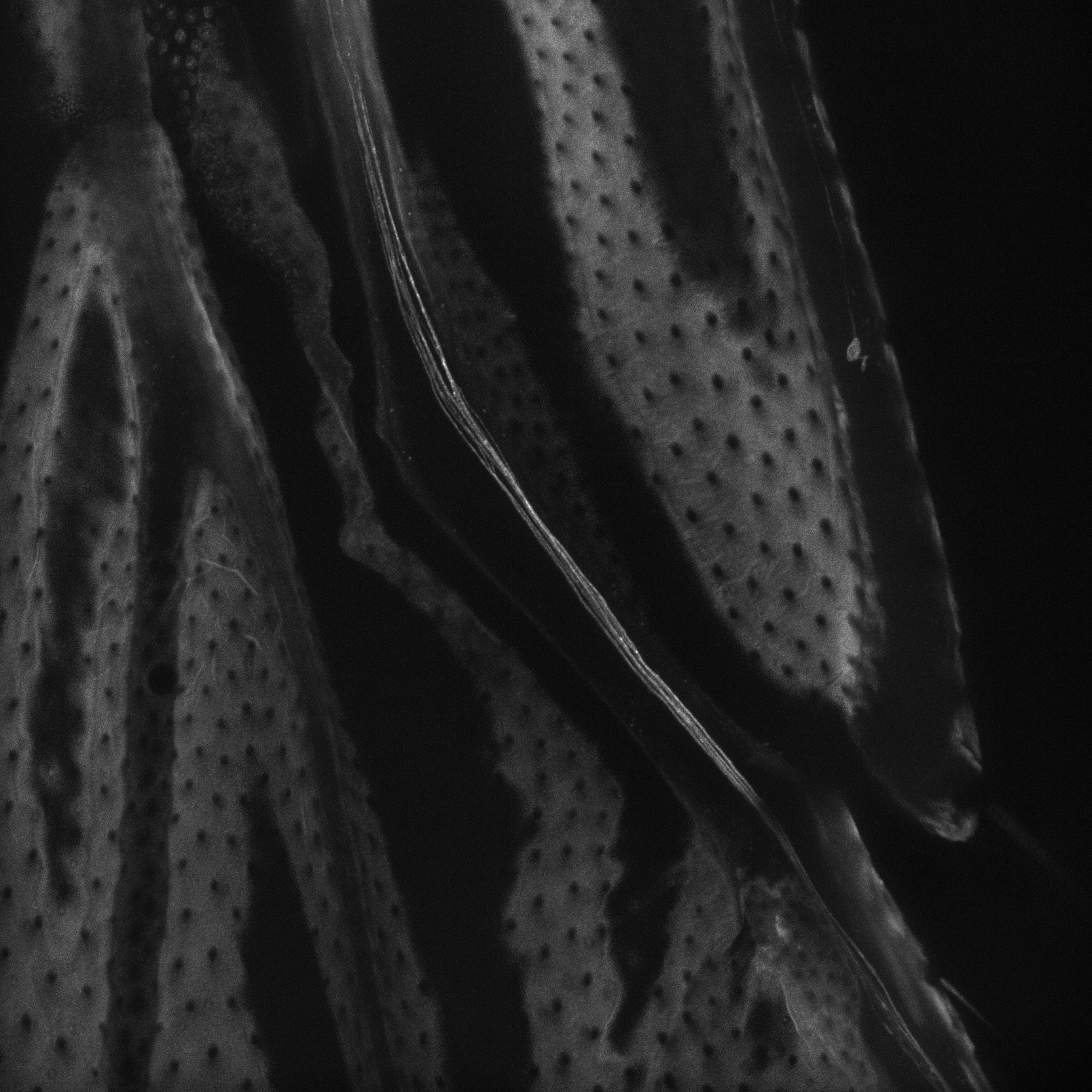

Supplement: Supplementary file 15 — Source data Fig. 1 [file 44319_2024_301_MOESM15_ESM.zip › Figure 1/1A/nmnatoe 1 week after injury.tif]

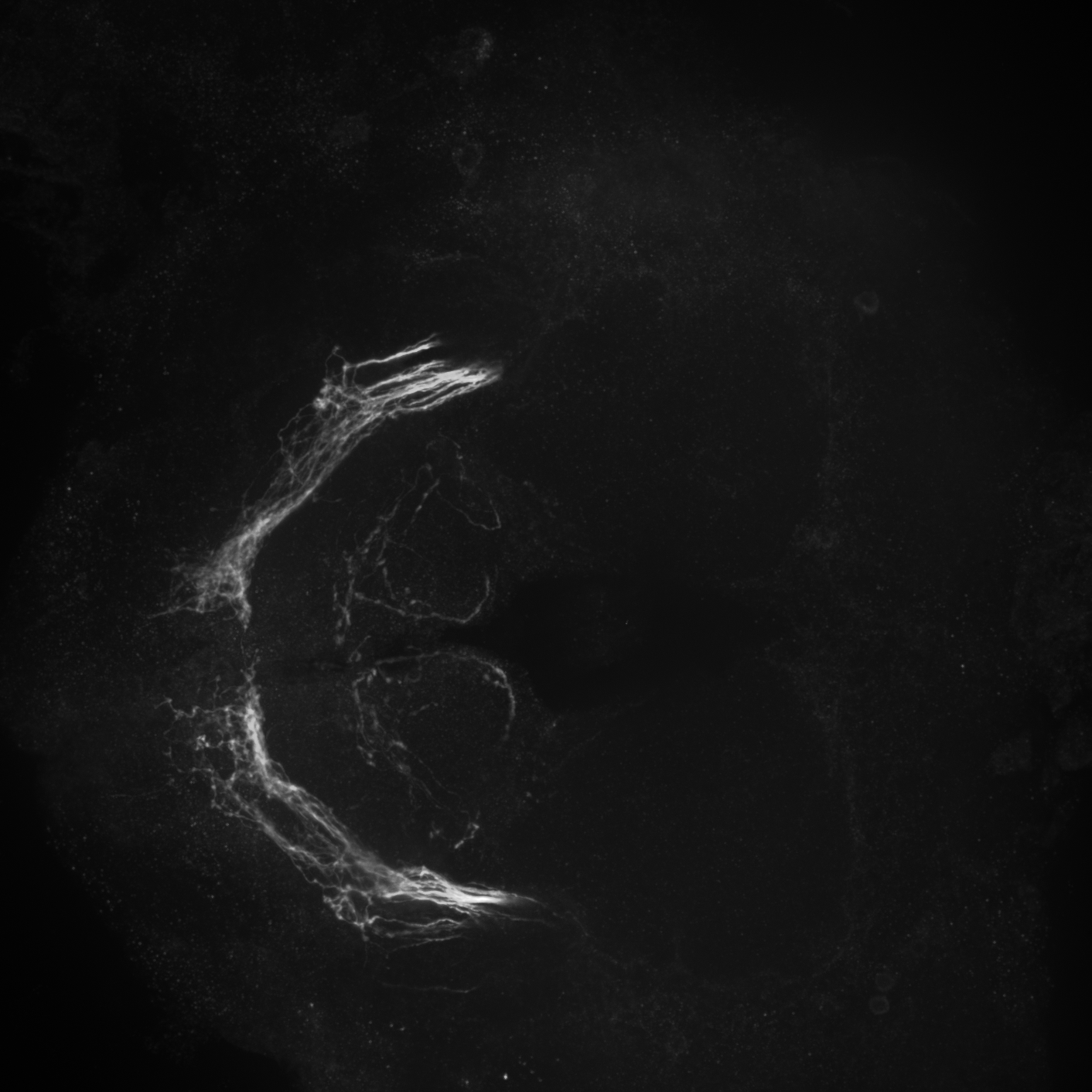

Supplement: Supplementary file 15 — Source data Fig. 1 [file 44319_2024_301_MOESM15_ESM.zip › Figure 1/1E/GFP-MAX_JOnmnat_before14_#7.tif]

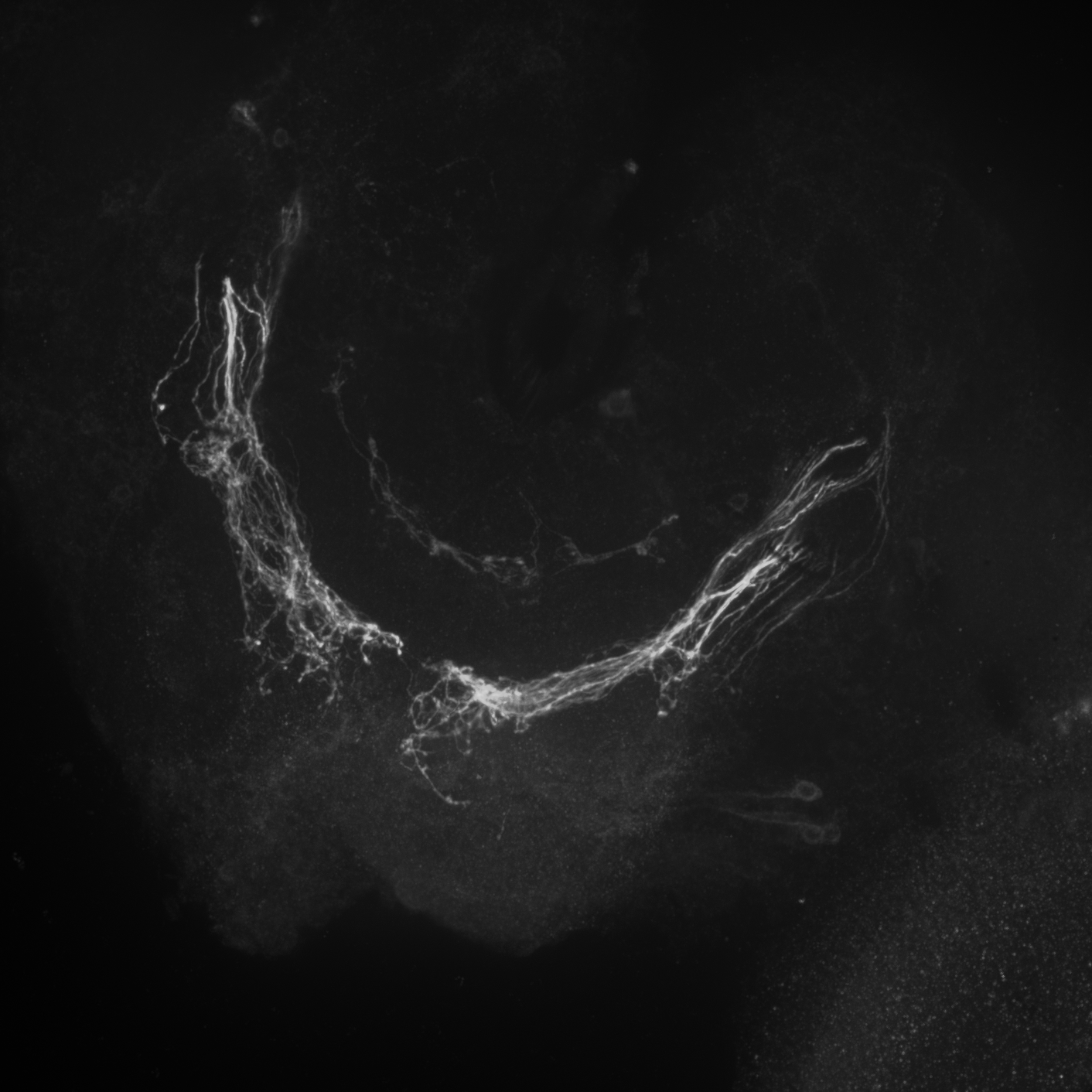

Supplement: Supplementary file 15 — Source data Fig. 1 [file 44319_2024_301_MOESM15_ESM.zip › Figure 1/1E/GFP-MAX_JO_wt_before14_3.tif]

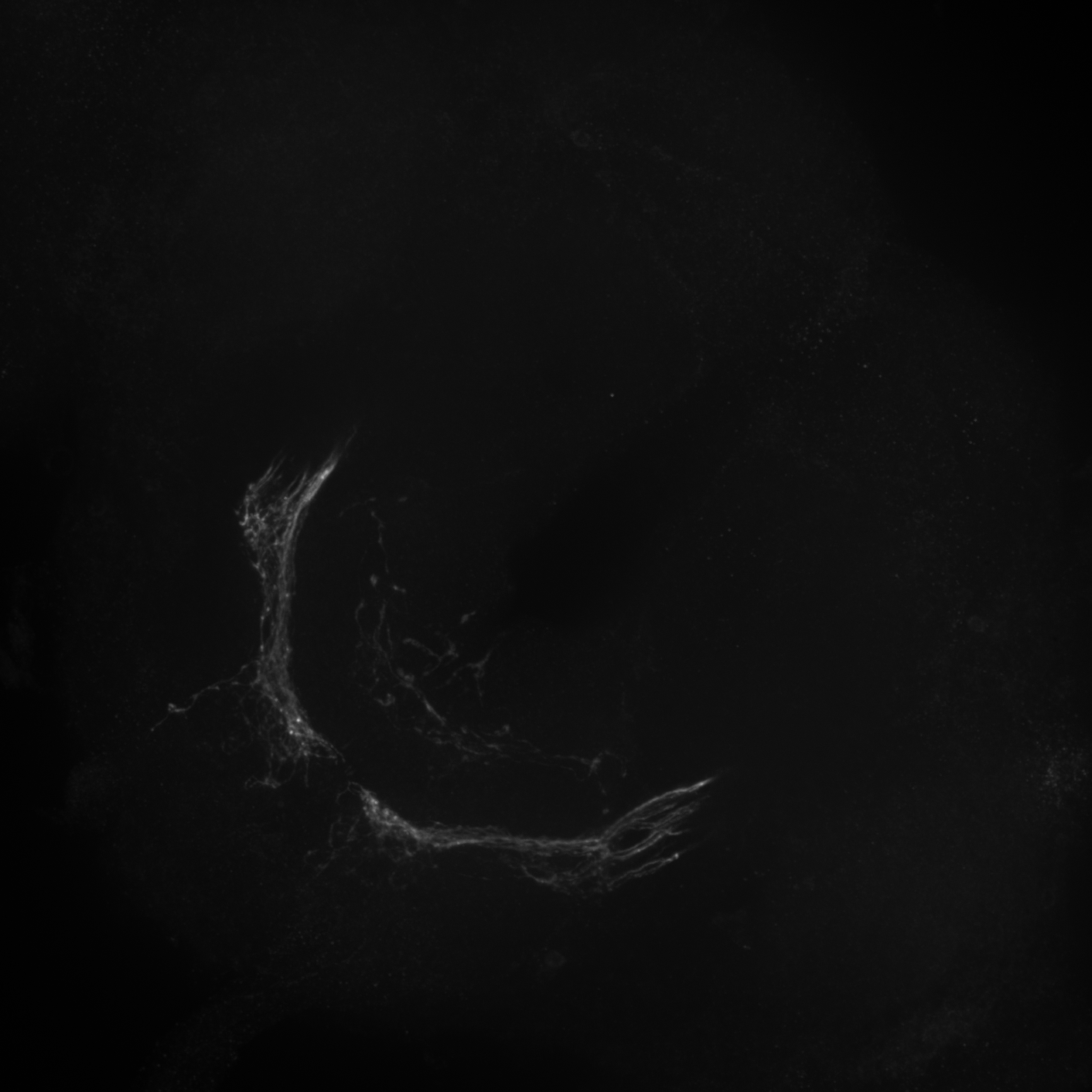

Supplement: Supplementary file 15 — Source data Fig. 1 [file 44319_2024_301_MOESM15_ESM.zip › Figure 1/1E/GFP-MAX_JOnmnat_14dpa_4.tif]

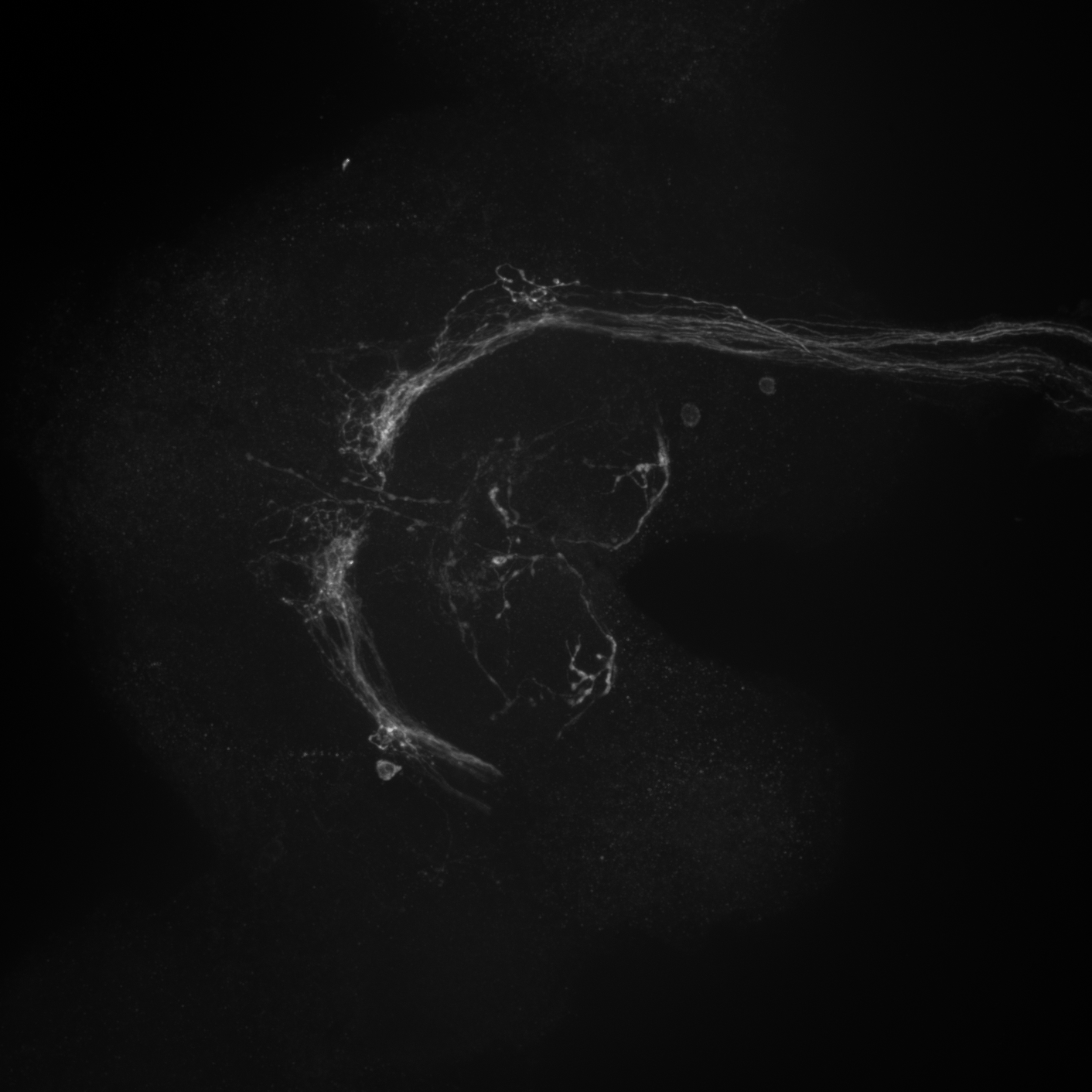

Supplement: Supplementary file 15 — Source data Fig. 1 [file 44319_2024_301_MOESM15_ESM.zip › Figure 1/1E/GFP-MAX_JOnmnat_7dpa__6-1.tif]

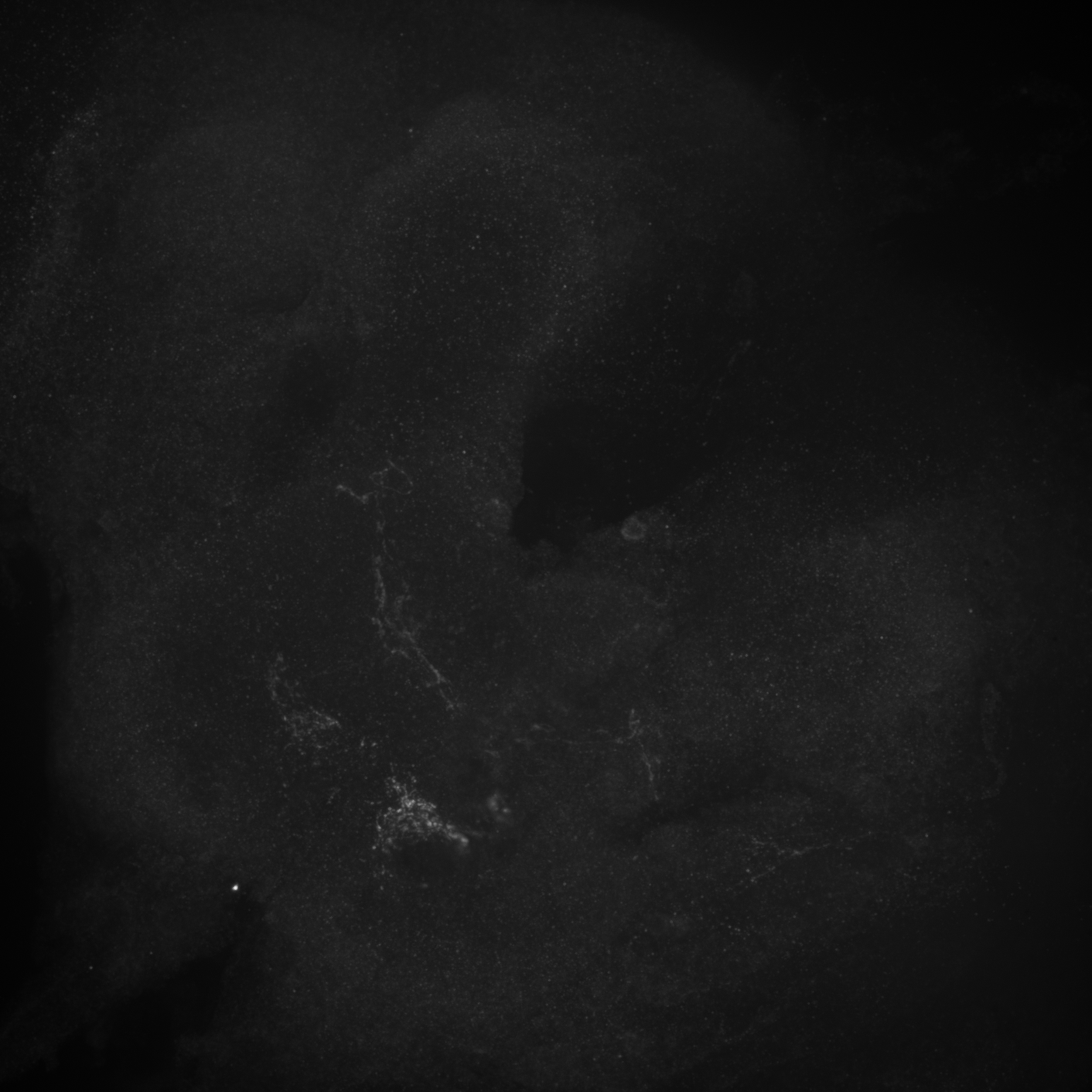

Supplement: Supplementary file 15 — Source data Fig. 1 [file 44319_2024_301_MOESM15_ESM.zip › Figure 1/1E/GFP-MAX_JOwt_7dpa_3_.tif]

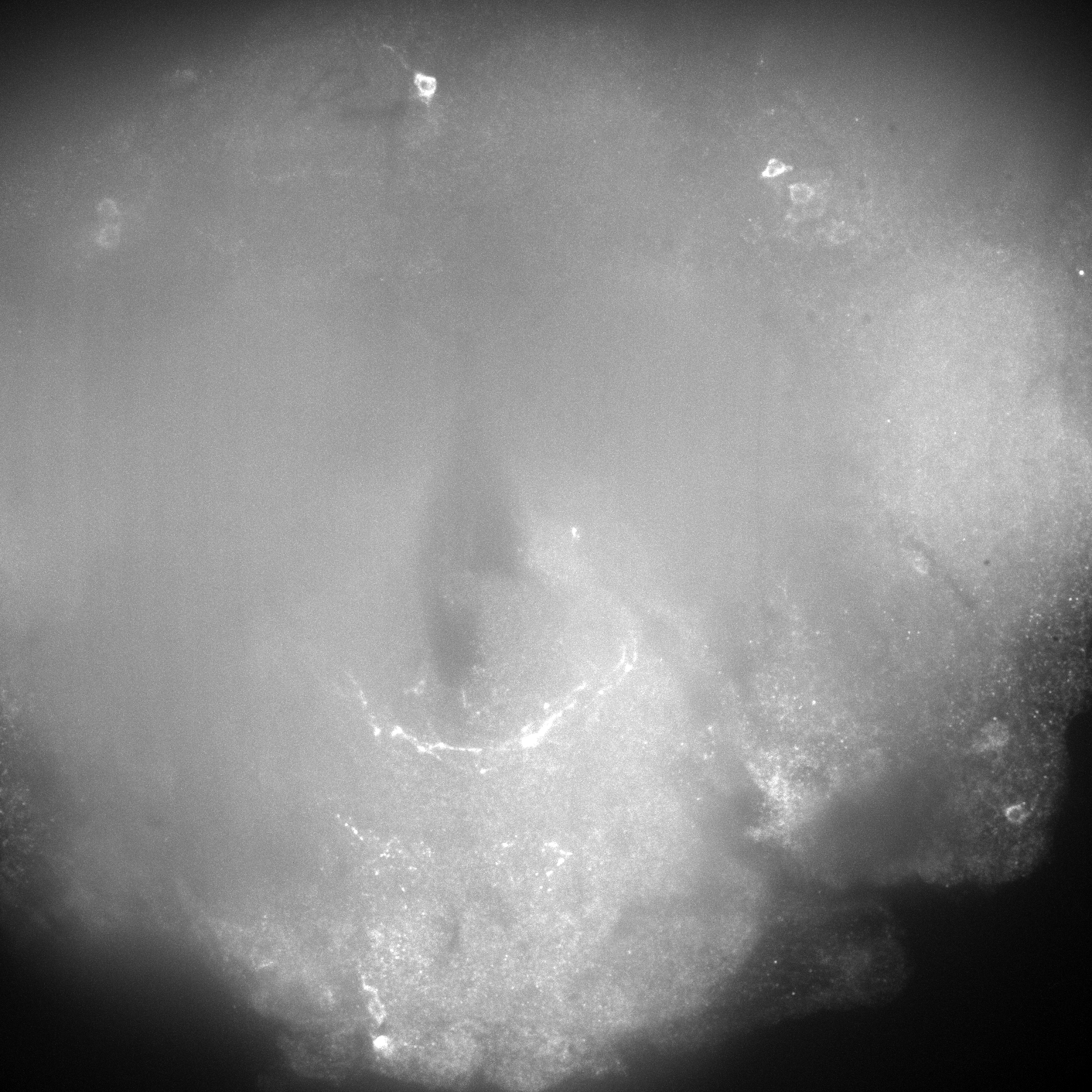

Supplement: Supplementary file 15 — Source data Fig. 1 [file 44319_2024_301_MOESM15_ESM.zip › Figure 1/1E/GFP-MAX_JOwt_14dpa_#1.tif]

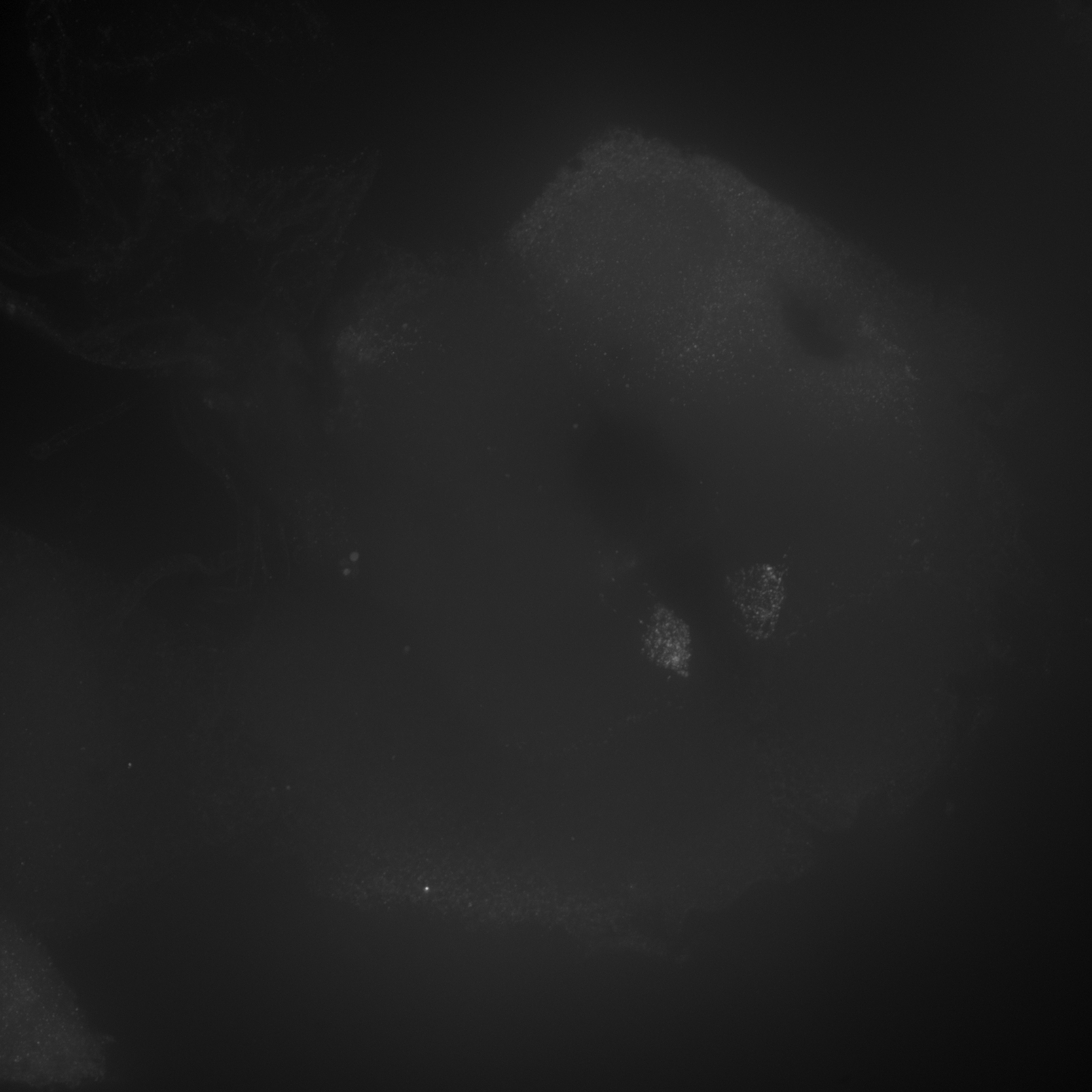

Supplement: Supplementary file 15 — Source data Fig. 1 [file 44319_2024_301_MOESM15_ESM.zip › Figure 1/1C/GFP-or22a_wt_14dpa_#2.tif]

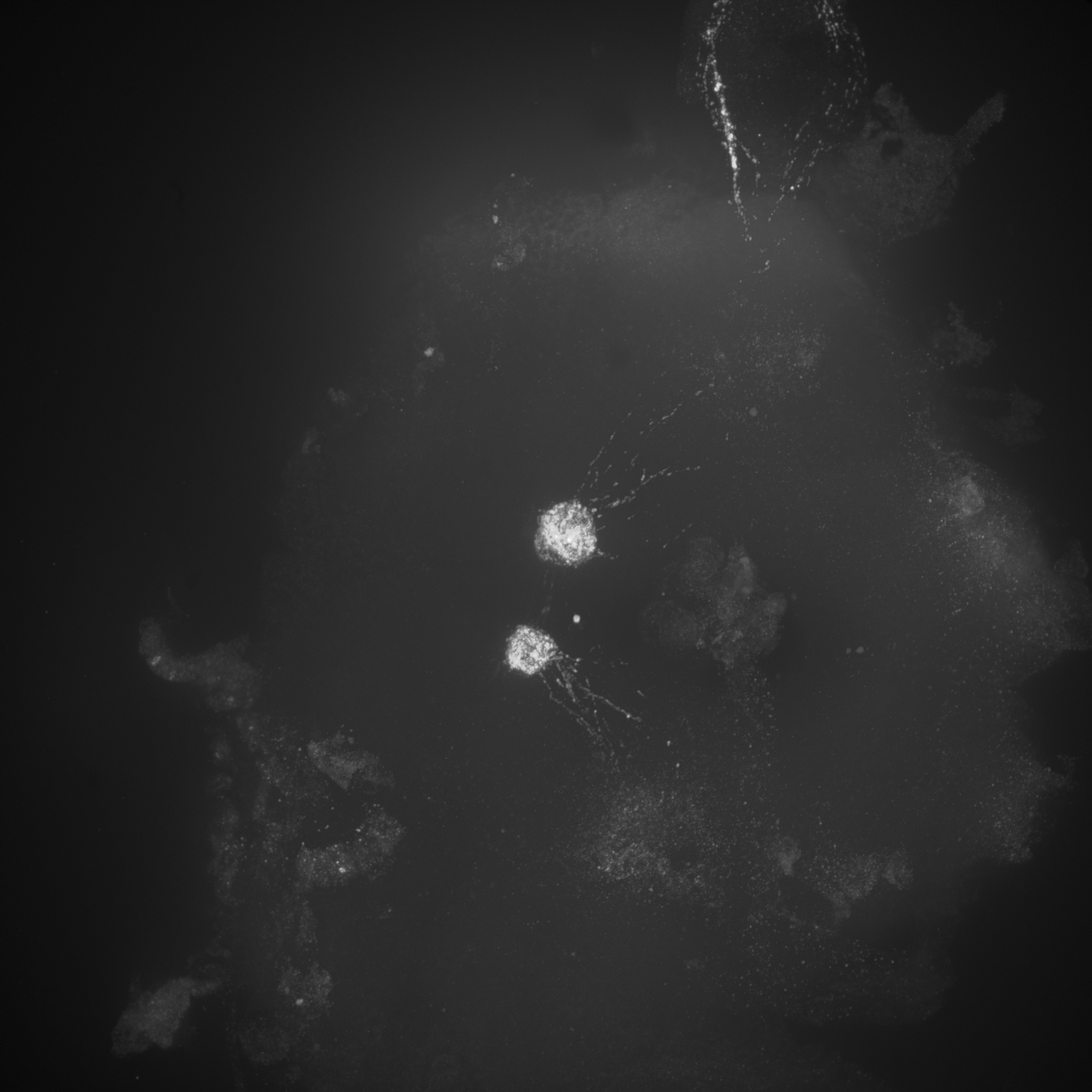

Supplement: Supplementary file 15 — Source data Fig. 1 [file 44319_2024_301_MOESM15_ESM.zip › Figure 1/1C/GFP-or22a_wt_7dpa__5.tif]

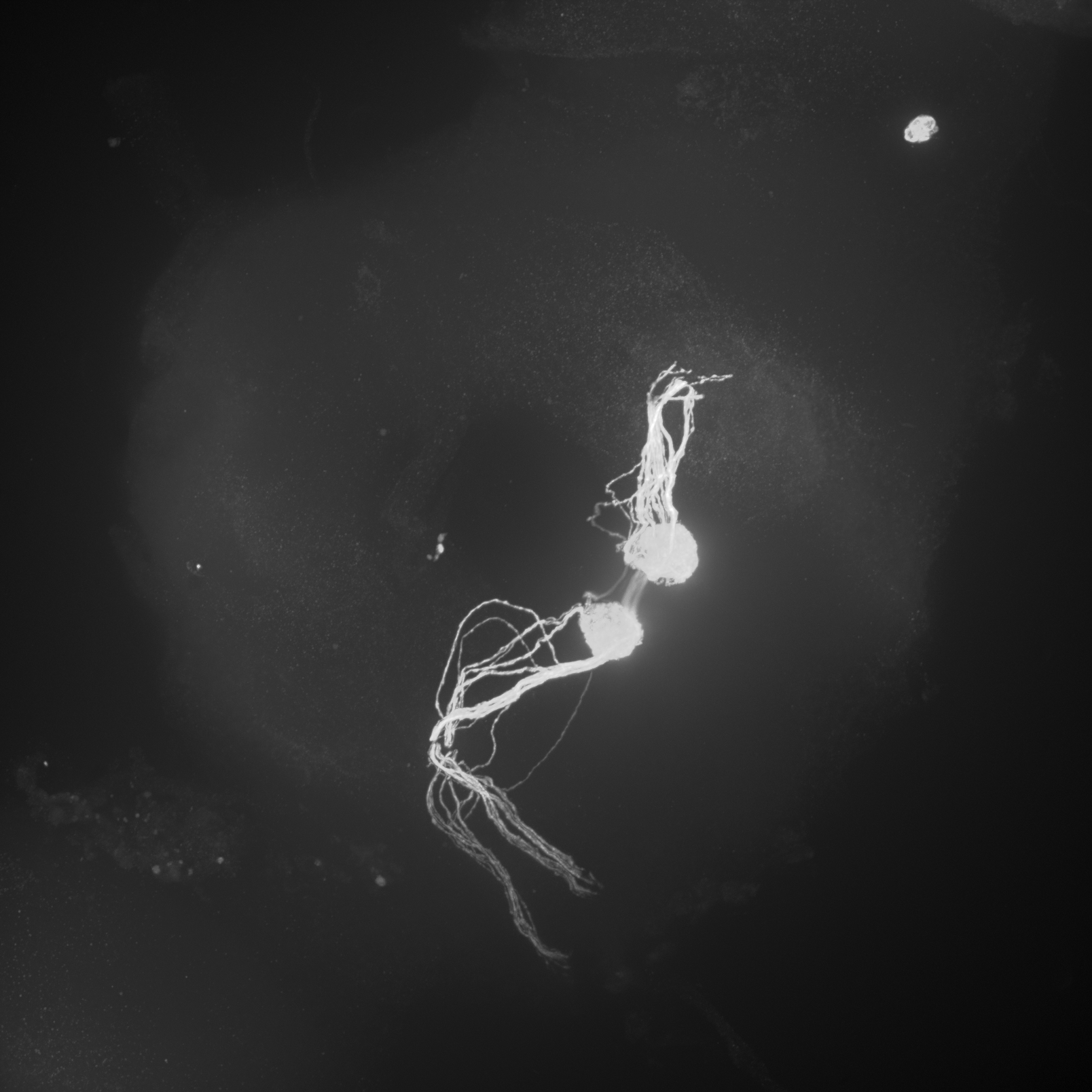

Supplement: Supplementary file 15 — Source data Fig. 1 [file 44319_2024_301_MOESM15_ESM.zip › Figure 1/1C/GFP-or22a_wt_before7__10.tif]

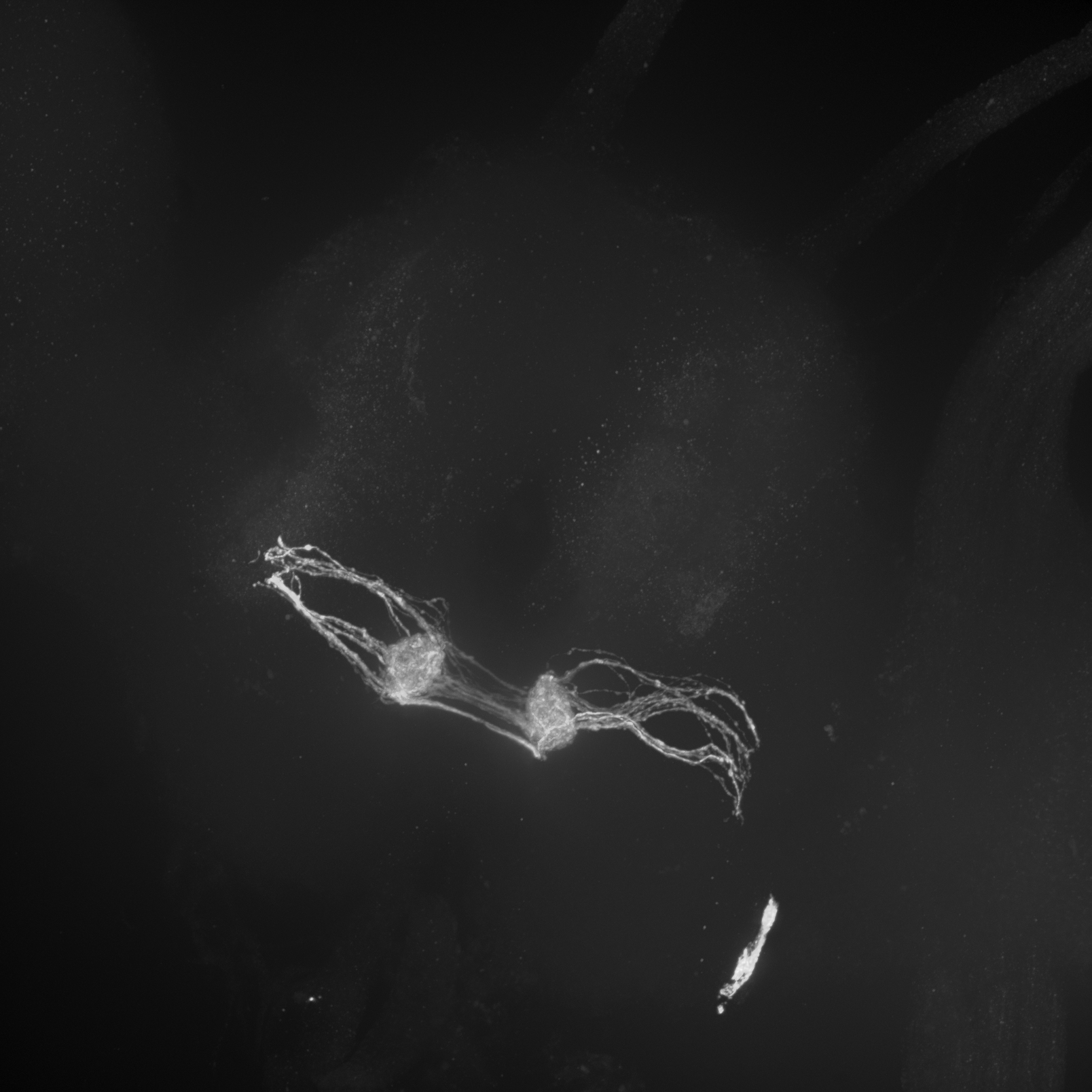

Supplement: Supplementary file 15 — Source data Fig. 1 [file 44319_2024_301_MOESM15_ESM.zip › Figure 1/1C/GFP-or22a_nmnat_7dpa_#4.tif]

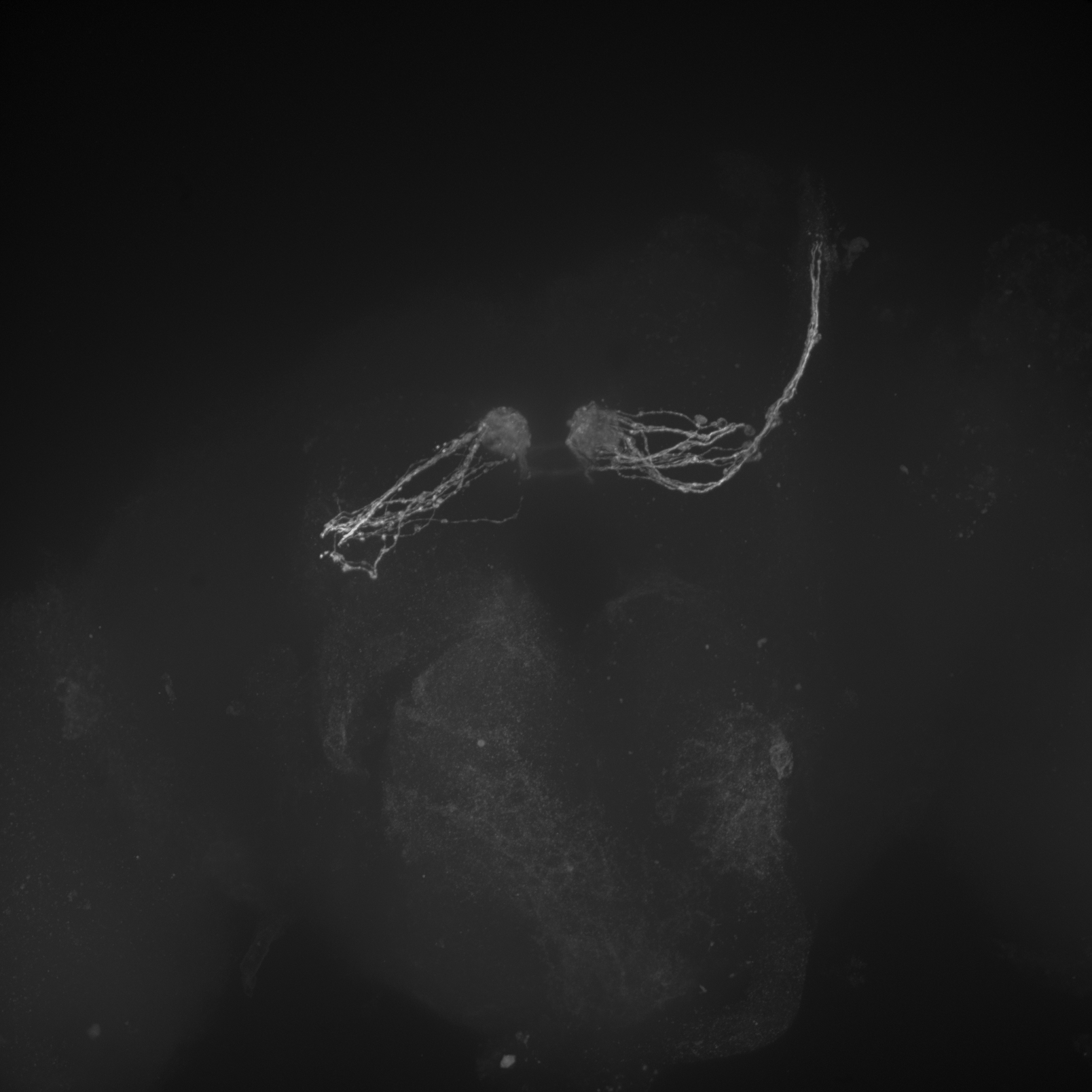

Supplement: Supplementary file 15 — Source data Fig. 1 [file 44319_2024_301_MOESM15_ESM.zip › Figure 1/1C/GFP-or22a_nmnat_14dpa_#3.tif]

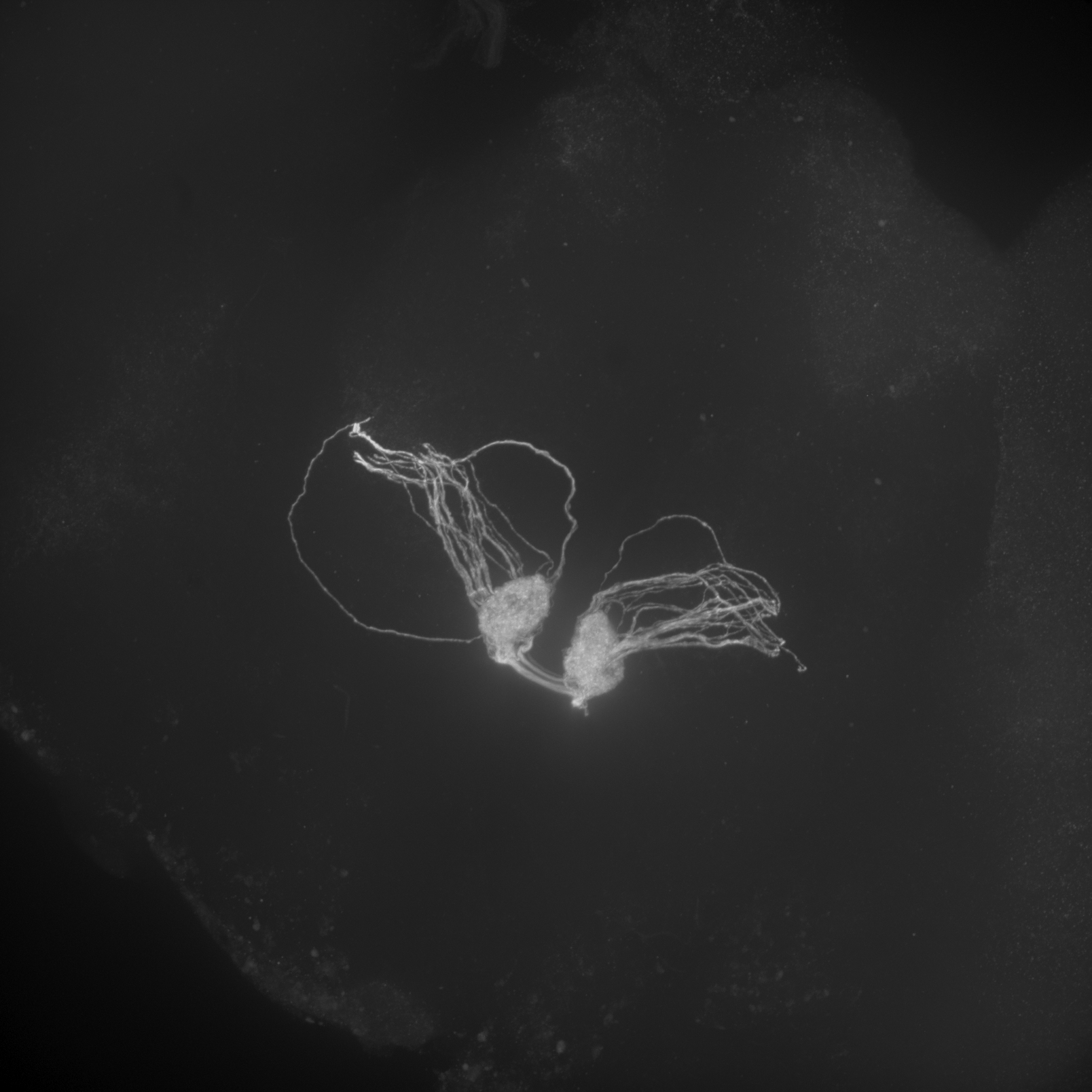

Supplement: Supplementary file 15 — Source data Fig. 1 [file 44319_2024_301_MOESM15_ESM.zip › Figure 1/1C/GFP-or22a_nmnat_before14_#7.tif]
