# Supplementary material for: Local translatome sustains synaptic function in impaired Wallerian degeneration
Source: EMBO Rep. 2024 Oct 31;26(1):61–83. doi: 10.1038/s44319-024-00301-8 (PMC11724096; doi:10.1038/s44319-024-00301-8)
Supplement: Supplementary file 21 — Expanded View Figures [file 44319_2024_301_MOESM21_ESM.pdf]

# Expanded View Figures

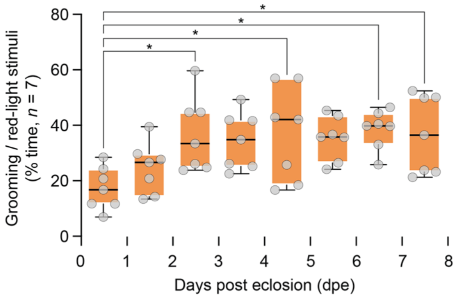


**Figure EV1. Time-course of optogenetically-evoked antennal grooming behavior in wild type after eclosion.**

Quantification of manually scored antennal grooming in wild-type flies with CsChrimson expressed in JO neurons between 0 to 7 days post eclosion (dpe). Data (% time of red-light stimuli; $n = 7$ animals), box (interquartile) and whisker (minimum and maximum, respectively) plot, with minimum, lower quartile (Q1), median, upper quartile (Q3), and maximum. One-way ANOVA with Tukey's multiple comparisons test. *$p < 0.05$.

© The Author(s)

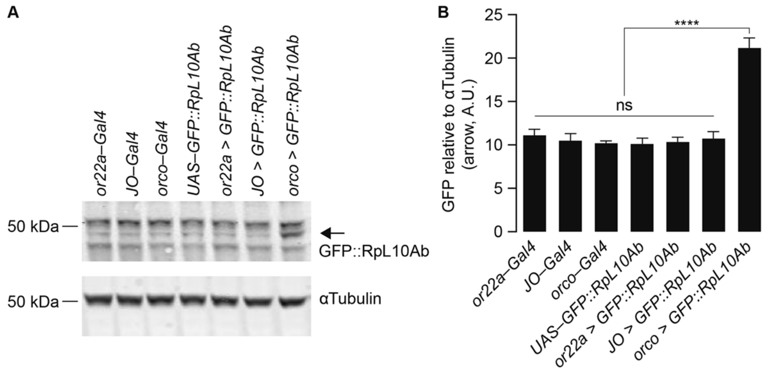


**Figure EV2. Olfactory organ projections as a large-scale source to express and detect GFP-tagged ribosomal protein 10Ab.**

(**A**) Western blot of *Drosophila* heads with GFP-tagged ribosomal protein 10Ab (GFP::RpL10Ab) expressed in *orco*$^{+}$ neurons. 4 heads/lane; arrow, molecular weight of GFP::RpL10Ab. (**B**) Quantification of GFP immunoreactivity by densitometry. Data, mean ± SEM ($n = 3$, three replicates of three biological experiments). Arbitrary units, A.U.; One-way ANOVA with Tukey's multiple comparisons test; ****$p < 0.0001$; ns (not significant), $p > 0.05$.

 © The Author(s)

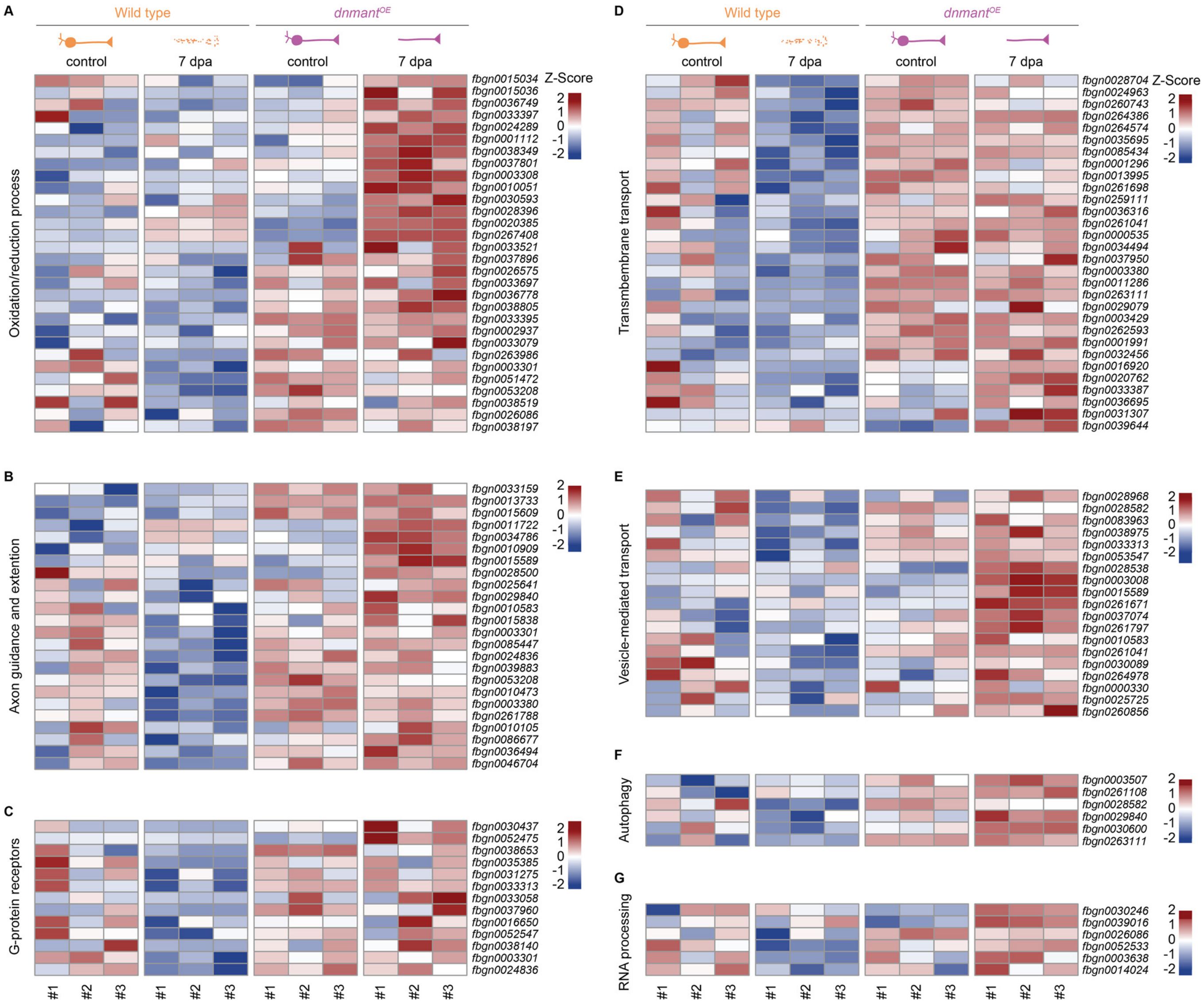


**Figure EV3. Heat maps of biological process GO terms significantly enriched in *dnmnat*$^{OE}$ 7 dpa.**

(**A**) Heat map of oxidation/reduction process transcripts ($n = 30$). (**B**) Heat map of axon guidance and extension transcripts ($n = 24$). (**C**) Heat map of G-protein receptor transcripts ($n = 13$). (**D**) Heat map of transmembrane transport transcripts ($n = 30$). (**E**) Heat map of vesicle-mediated transport transcripts ($n = 19$). (**F**) Heat map of autophagy transcripts ($n = 6$). (**G**) Heat map of RNA processing transcripts ($n = 6$). Expression levels, Z-Score average.

© The Author(s)

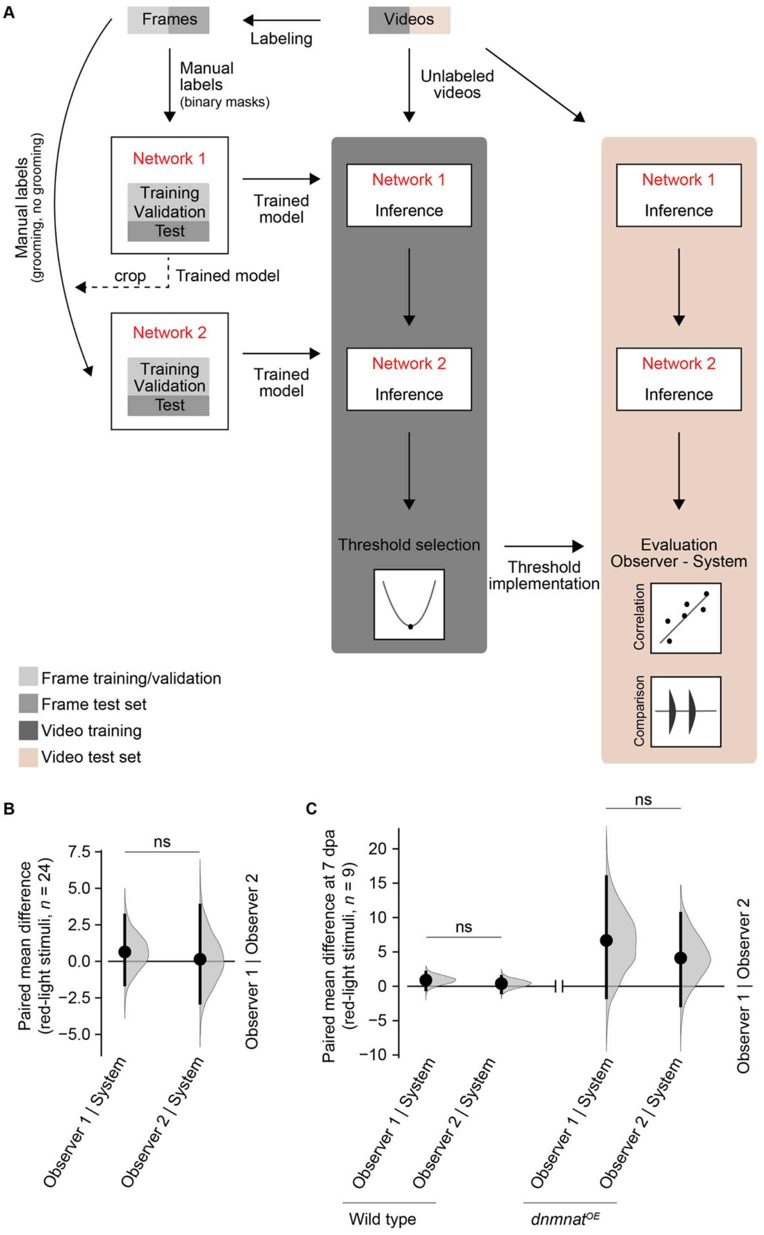


**Figure EV4. Dual-network training and evaluation pipeline for automated grooming detection and comparison with observer scores.**

(**A**) Illustration of Network 1 and 2 training and testing pipelines. Raw videos were split into training and test sets (gray and red, respectively). Video frames from the training set were manually labeled to identify the position of the head in the image (e.g., binary mask) and assign the grooming or no grooming labels. Network 1 was trained, validated, and tested on its own training set (frames). Network 2 was trained, validated, and tested on the labeled frames cropped by Network 1. Both networks, once trained, were used to determine the optimal threshold value of grooming detection using the same training set (raw videos). Finally, the test set (raw videos) was used to evaluate the accuracy of the system by comparing its performance with the scores of the observer. (**B**) Standard deviation between grooming scores given by each observer to the same uninjured wild-type animal ($n = 24$ animals). (**C**) Standard deviation between grooming scores given by each observer to the same wild type and *dnmnat*$^{OE}$ at 7 dpa ($n = 9$ animals). The line break denotes the separation between the controls for each genotype. The plots illustrate the similarity between the standard deviations of observer 1 versus the system and observer 2 versus the system, as compared to the standard deviation observed among observers (horizontal black line, mean, number of frames). Non-parametric one-way ANOVA (Friedman test) with Dunn multiple comparisons test; ns, $p > 0.05$.


© The Author(s)

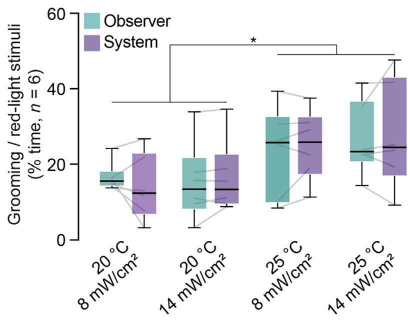


**Figure EV5. Detection of temperature-dependent changes in antennal grooming.**

Quantification of antennal grooming detection by observer and system in wild-type flies with CsChrimson expressed in JO neurons of 7-day-old animals (green and purple, respectively; probability, ≥0.3; % time of red-light stimuli; $n = 6$ animals). Data, box (interquartile) and whisker (minimum and maximum, respectively) plot, with minimum, lower quartile (Q1), median, upper quartile (Q3), and maximum. Three-way ANOVA; $*p < 0.05$.
